# Enhancing Sex Estimation Accuracy with Cranial Angle Measurements and Machine Learning

**DOI:** 10.3390/biology13100780

**Published:** 2024-09-29

**Authors:** Diana Toneva, Silviya Nikolova, Gennady Agre, Stanislav Harizanov, Nevena Fileva, Georgi Milenov, Dora Zlatareva

**Affiliations:** 1 Institute of Experimental Morphology, Pathology and Anthropology with Museum, Bulgarian Academy of Sciences, 1113 Sofia, Bulgaria; sil_nikolova@abv.bg; 2 Institute of Information and Communication Technologies, Bulgarian Academy of Sciences, 1113 Sofia, Bulgaria; gennady.agre@iict.bas.bg (G.A.); sharizanov@parallel.bas.bg (S.H.); 3 Faculty of Medicine, Medical University of Sofia, 1431 Sofia, Bulgaria; nfileva@medfac.mu-sofia.bg (N.F.); g.milenov@medfac.mu-sofia.bg (G.M.); dorazlat@medfac.mu-sofia.bg (D.Z.)

**Keywords:** sex estimation, cranial angles, machine learning, classification models

## Abstract

**Simple Summary:**

Sex estimation based on bones is a technique used to determine the biological sex of an individual from skeletal remains. It relies on the anatomical differences between male and female skeletons. Various bone characteristics have been incorporated into methods for sex estimation. Linear measurements are commonly used features in classification models for sex estimation. On the other hand, angle measurements are rarely included in such models, although they are important characteristics of the geometry of the bones and could provide essential information for the discrimination between the male and female bones. The goal of this research is to examine the potential of cranial angles for sex estimation and to identify the set of the most dimorphic angles by applying machine learning algorithms.

**Abstract:**

The development of current sexing methods largely depends on the use of adequate sources of data and adjustable classification techniques. Most sex estimation methods have been based on linear measurements, while the angles have been largely ignored, potentially leading to the loss of valuable information for sex discrimination. This study aims to evaluate the usefulness of cranial angles for sex estimation and to differentiate the most dimorphic ones by training machine learning algorithms. Computed tomography images of 154 males and 180 females were used to derive data of 36 cranial angles. The classification models were created by support vector machines, naïve Bayes, logistic regression, and the rule-induction algorithm CN2. A series of cranial angle subsets was arranged by an attribute selection scheme. The algorithms achieved the highest accuracy on subsets of cranial angles, most of which correspond to well-known features for sex discrimination. Angles characterizing the lower forehead and upper midface were included in the best-performing models of all algorithms. The accuracy results showed the considerable classification potential of the cranial angles. The study demonstrates the value of the cranial angles as sex indicators and the possibility to enhance the sex estimation accuracy by using them.

## 1. Introduction

Skeletal remains provide valuable information about various biological characteristics of an individual (including sex, age, ancestry, stature, etc.). The extraction of this information can be achieved by different features and approaches. The use of macroscopic traits is considered a straightforward method for obtaining data about an individual’s biological profile. The methods based on measurable characteristics are also widely used in anthropological studies because of their quantifiable nature and potential for yielding essential information after adequate statistical processing. Linear measurements are established as standard metrics in morphometric studies because they can be easily obtained directly and virtually. Angles are particularly useful for the metric description of complex structures like the facial skeleton, pelvic bones, and long bone epiphyses, but they are frequently overlooked due to the difficulties in measuring them, which requires specialized equipment. Although the use of virtual tools facilitates their measuring, they are often omitted from the studies. However, angles could provide valuable information about bone morphology regardless of size differences between compared objects.

The human skull is the most complex part of the axial skeleton and is a focus of many studies due to its unique assemblage of bones and the variation observed in its components in relation to developmental, evolutionary, genetic, and environmental influence. Moreover, the skull has been examined because of its connection to the brain, sensory organs, onset of the respiratory and digestive systems, facial soft tissues and, in particular, its association to the facial appearance. In forensic anthropology, cranial measurements have been extensively used, particularly in sex estimation studies, where they are incorporated into models for sex classification. Cranial angles have been included in several sex estimation models along with linear measurements, making it difficult to distinguish their individual contribution [1,2,3,4,5]. The studies based solely on cranial angles have either focused on a specific bone, such as the frontal bone [6,7], or on a particular segment, such as the nasion-glabella-sella triangle [8]. In the referenced studies, the angular values have been obtained using different types of images: cephalograms [1,2,4,8], three-dimensional (3D) models [3,5], and two-dimensional (2D) lateral screenshots on 3D models [6,7].

Various imaging technologies can be employed to generate images of bones, including medical imaging of living individuals, as well as laser scanning and photogrammetry of dry bone specimens. Working with images facilitates not only the easy calculation of traditionally used measurements but also the derivation of additional metrics that would be difficult to obtain through direct measurement methods. This is particularly useful for angles that fall into three categories: between two vectors, between a vector and a plane, and between two planes. Thus, their calculation requires between three and six points. Some studies use geometric morphometrics to differentiate male and female skulls based on shape, but they examine larger cranial regions and provide a generalized overview of sex differences in landmark configurations [9,10,11,12,13]. Yet, the use of metric data offers a more straightforward approach for incorporating these measurements into sex estimation models.

Sex estimation based on bone features is a standard classification task in forensic anthropology. Machine learning (ML) algorithms are proper tools for classifying input data into mutually exclusive categories, and so far, they have been extensively used to develop sex estimation models. Various classification algorithms have been applied for that purpose, as most of them belong to the type of eager learners such as support vector machines (SVM), artificial neural networks, naïve Bayes (NB), and decision trees [14,15,16,17,18,19,20,21]. The advantage of the ML algorithms that are eager learners is that the intensive computation is performed during the training phase while building a generalized prediction model, and its application afterwards is very fast, providing quick predictions. Since classification is a form of supervised learning, the input data used for training ML models should be clearly assigned to specific classes. In studies providing sex estimation models, the classes are “male” and “female”, and these labels need to be derived from trustworthy sources. This would guarantee the development of models allowing fast sex identification in current cases.

The present study aimed to assess the usefulness of cranial angles for sex estimation and to differentiate the subset of most dimorphic angular characteristics. For this purpose, a set of ML algorithms was trained on datasets containing only cranial angles to evaluate the accuracy of the models for sex estimation and to identify the angles involved in the best-performing classifiers, i.e., those providing the most accurate sex identification.

## 2. Material and Methods

Computed tomography (CT) scans of 334 adult Bulgarians (154 males and 180 females) were used in the study. The mean age of the males was 54 years (age range: 19–89 years) and that of the females was 57 years (age range: 20–89 years). The head scanning was performed by a medical CT system Toshiba Aquilion 64. The scanning parameters included a detector configuration of 32 × 0.5 mm, a tube voltage of 120 kV, a tube current in the range of 165 to 500 mA, and an exposure time of 0.5 s. The reconstruction parameters were set as follows: a reconstruction matrix of 512 × 512 pixels, a slice thickness of 0.5 mm, a reconstruction interval of 0.3 mm, and the convolution filter FC63. The study was approved by the Human Research Ethics Committee at the IEMPAM-BAS (No. 14/21.02.2022). All DICOM series were anonymized, keeping only the information about the sex and age of the individuals. The CT images included in the study did not show any visible pathological changes on the skull bones. The DICOM series were used to generate polygonal models of the skulls in InVesalius 3.1. (CTI, São Paulo, Brazil). This procedure included segmentation of the bone tissue at a predefined threshold of 227-3071 HU and saving of the produced surface models in STL format. Afterwards, the 3D coordinates of 37 landmarks were collected from the skull models using MeshLab 2016.12 [22] (Table 1).

**Figure 1:**
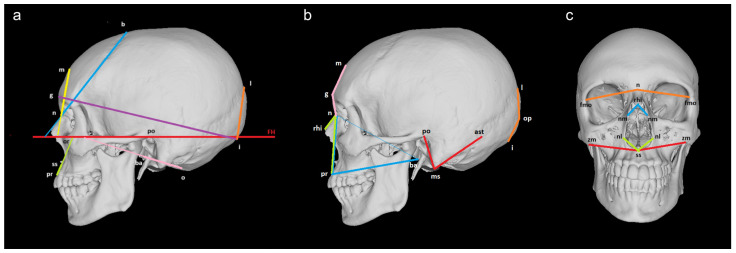
Cranial angles measured between (**a**) a line and a plane: n-b-FH (blue), n-m-FH (yellow), g-i-FH (purple), l-i-FH (orange), ss-pr-FH (green), ba-o-FH (pink); (**b**) two lines (lateral view): n-g-m (pink), n-pr-ba (blue), rhi-n-pr (green), l-op-i (orange), po-ms-ast (red); (**c**) two lines (front view): fmo-n-fmo (orange), nm-rhi-nm (blue), nl-ss-nl (green), zm-ss-zm (red).

Thirty-six angles were calculated based on the 3D coordinates of the cranial landmarks (Table 2). Two types of angles were computed: between a line and a plane and between two lines (Figure 1, Supplementary File S1). Twenty (14 midsagittal and 3 bilateral) angles referred to the first type, which was calculated based on the direction vector of the line and the normal vector of the plane i.e., the arccosine of the ratio of the dot product of the two vectors to the product of their magnitudes. The plane in all these cases represented the Frankfurt horizontal plane (FH) built between the landmarks left orbitale, left porion, and right porion. The other sixteen (9 midsagittal, 5 transversal, and 1 bilateral) angles represented the second type of angles. The calculation of these angles was based on the Law of Cosines and the sides’ lengths of the triangle constructed for each set of three landmarks. The triangle’s sides were calculated as interlandmark distances in PAST, version 2.17c [23]. The computation of the angles was performed in MATLAB 2018a and MS Excel 2010.

The normality in the data distribution of each angle was assessed by the Shapiro–Wilk test and the equality of variances of the two samples was assessed by the Levene’s test. Depending on the results of these two tests, the significance of the sex differences was evaluated using either the t-test or the Mann–Whitney U-test. The significance level was set at *p* < 0.05.

Intraobserver errors were assessed by calculating the standard deviation of acquisition trials for each landmark [24]. The landmarks were digitized three times on 40 crania. There was a gap of 3 to 5 days between the successive trials. The error results of the neurocranial landmarks were reported in Toneva et al. [12] and those of the viscerocranial landmarks were described in Toneva et al. [13]. A summary of the errors of the landmarks included in the present study is provided in Table S1. All of them showed small intraobserver errors within 1 mm.

Several ML algorithms were selected for learning classification models (classifiers) for sex estimation. The algorithms are as follows: the rule induction algorithm CN2 [25], the optimization algorithm Support Vector Machines (SVM) [26], the probabilistic naïve Bayes (NB) [27] and logistic regression (LR) [28]. All these algorithms were implemented in the machine learning and data visualization environment Orange 2.7.8. The default values provided by the Orange environment were used for all parameters of the algorithms. SVM was applied with the RBF kernel, and the other parameters of the algorithm were set only once before the first run of cross-validation using the option “Automatic parameter search” in the Orange environment.

The training datasets of this study included examples belonging to two classes: males (class 1) and females (class 2). The classification accuracy of the default (majority-based, MB) classifier was 0.539. The ML classification models were evaluated by applying a repeated stratified k-cross-validation schema [29]. A 5-fold cross-validation, repeated 10 times at different randomly selected initial conditions, was used. Three measures were used to evaluate the quality of the models: average classification accuracy (the proportion of all correctly classified examples averaged across the 10 runs of the 5-fold cross-validation), and class-dependent classification accuracy for each class (the proportion of correctly classified examples belonging to class 1 or class 2 averaged across all runs of the 5-fold cross-validation). All classifiers were trained and tested on the same data subsets constructed by the repeated 5-fold cross-validation.

An attribute selection scheme was applied for selecting the most important attributes to train the ML algorithms and enhance their classification ability. The Correlation-based Feature Selection method [30] implemented as the CfsSubsetEval algorithm in the Weka environment (https://ml.cms.waikato.ac.nz/weka/index.html, accessed on 8 September 2024) was used as an attribute-selection method (ASM). This filter approach evaluates the quality of a subset of attributes by considering the individual predictive ability of each attribute along with the degree of redundancy between them. For searching the best subset of attributes by this method, the Weka BestFirst algorithm [31] was applied. It searches the space of attribute subsets by greedy hillclimbing augmented with a backtracking facility. Firstly, the selected ASM was applied in 5-fold cross-validation mode, and thus, the attribute selection was done based on five different subsets of the training sets, each containing only 80% of the training examples. This step was repeated 10 times with randomly selected initial conditions in order to avoid the results being dependent on the initial selection of examples included in data subsets. Hence, fifty different subsets of attributes were constructed for each training dataset. Secondly, the frequency of each attribute selection by ASM was calculated. The more frequently an attribute was selected, the more important it was for the sex classification. Finally, the attributes were grouped into several subsets, which corresponded to specific attribute importance (AI) intervals (e.g., >0, >0.1, >0.2; >0.3, etc.). Each ML algorithm was applied to all attribute subsets to identify the one that achieved the highest classification accuracy.

## 3. Results

### 3.1. Sex Differences

Significant sex differences were established in 27 out of the 36 cranial angles i.e., in 75% of all angles (Table 3). They described different parts of the skull, such as the frontal bone, occipital bone, mastoid process, nasal bones, and nasal aperture, and facial convexity. However, non-significant sex differences were observed in some standard angles, such as the frontal slope angle, facial profile angle, nasal profile angle, nasal bone slope angle, alveolar profile angle, etc.

### 3.2. ML Classifiers

The initial dataset used to train the ML algorithms comprised the cranial angles that exhibited significant sex differences. All ML algorithms trained on this dataset of 27 cranial angles produced classification accuracy of more than 80%. The best result was achieved by SVM (91.3%), while the lowest result was obtained by the CN2 rules (81.7%).

**Figure 2:**
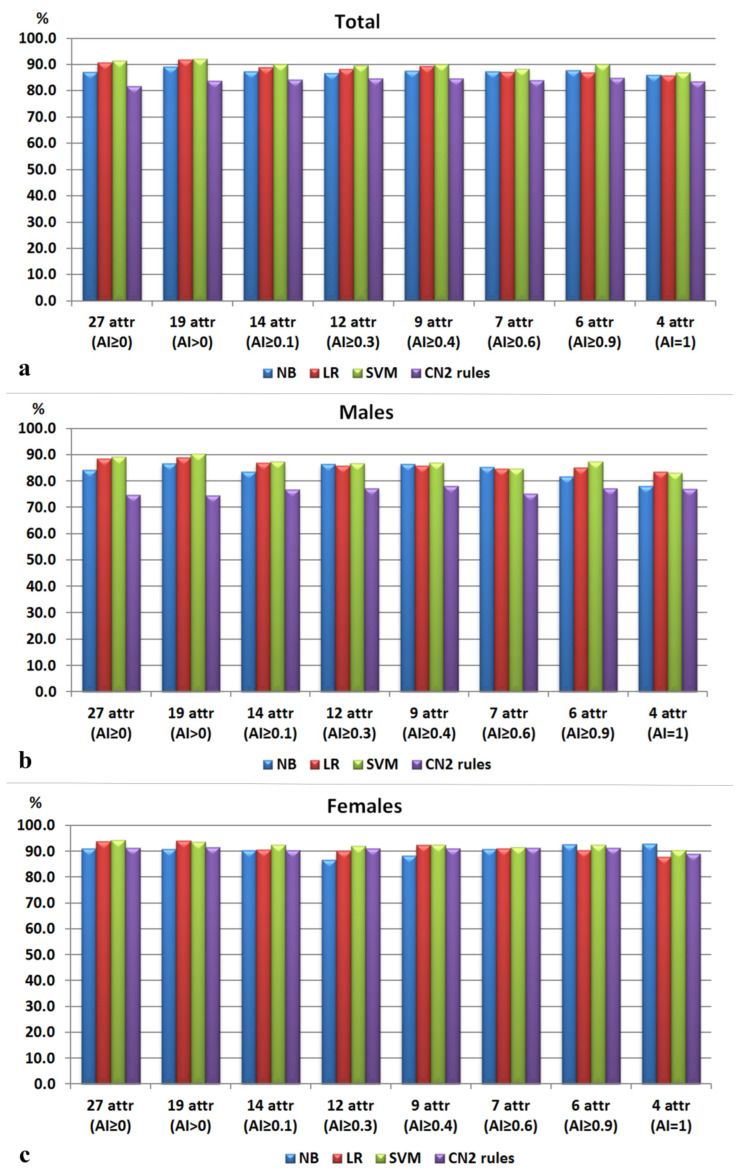
Accuracy of the models for sex estimation: (**a**) for the whole sample; (**b**) for class 1 (males); (**c**) for class 2 (females).

**Figure 3:**
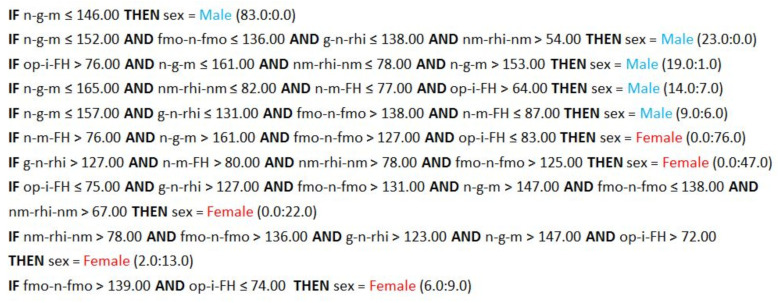
CN2 rules based on the attribute subset with AI ≥ 0.9. The numbers in brackets following each rule indicate its coverage. The first number indicates how many examples from the training set are classified correctly to class 1 by this rule, while the second number represents the number of examples from the training set that are misclassified by the rule.

Implementing the attribute selection scheme led to improved accuracy rates across all ML algorithms. The attributes included in each selection dataset are given in Table 4. The dataset including the attributes with AI > 0 provided the best accuracy for SVM, LR, and NB of all experiments (Figure 2, Supplementary File S2). This subset included nineteen features and represented a mixture of neurocranial and viscerocranial angles that described the upper and lower midface, the frontal, mastoid, and occipital regions, as well as their interrelations. The highest accuracy rate was attained by SVM (92.1%), closely followed by LR (91.8%), and finally by the NB model whose accuracy did not surpass 90%. The performance of the CN2 algorithm also improved when it was trained on the aforementioned dataset (AI > 0), but the highest accuracy for this algorithm was achieved with the dataset containing attributes with AI ≥ 0.9 (85%). Figure 3 presents a set of 10 rules generated by the CN2 algorithm, each covering at least 15 examples. Five of these rules described the class of males, and the other five described the class of females. The six attributes included in this subset (AI ≥ 0.9) and, respectively, in the CN2 rules, characterized the lower forehead region (n-g-m, n-m-FH, g-n-rhi), the upper midface (fmo-n-fmo, nm-rhi-nm), and the occipital protrusion at the inion (op-i-FH).

## 4. Discussion

Most previous morphometric studies on cranial sexual dimorphism have been focused on linear measurements and rarely made use of angular characteristics. Therefore, the present study examined whether angles are useful for sex estimation or their neglect in the former studies was justified.

In the studies conducted so far, the number and selection of investigated cranial angles have varied considerably, leading to inconsistent findings in observed significant sex differences [1,2,3,4,5,6,7,8,32]. The full set of cranial angles used in the present study has shown that three-quarters of them differ significantly between the male and female skulls. When cranial linear measurements have been compared, there is an ultimate prevalence in favour of the male crania because of the bigger size compared to that of the female crania [1,2,3,19,33,34,35,36,37,38,39]. However, the angles are size-irrelevant, and an angle can have the same value in a smaller and in a bigger skull. Thus, since the angles describe the shape of the geometric object independent of size, the presence of significant sex differences in cranial angles refers to the presence of significant differences in the geometry of the male and female skulls. The angles included in the models with the highest accuracy in the present study describe mainly cranial structures that are well-known to differ in shape between males and females (glabella, occipital protuberance, mastoids, nasal aperture, etc.). The lack of significant sex differences in some angles suggests that the corresponding special interactions between the defined lines and surfaces are consistent across skulls, regardless of sex.

The ML models developed in this study have shown that cranial angles are useful features for sex estimation identifying sex correctly in over 90% of the skulls. Such high accuracy has been reached by ML models using linear cranial measurements [19]. The standard discriminant and regression models based on linear measurements derived from different population groups have provided accuracy rates mostly in the range between 80% and 90% [3,33,34,35,36,37,38,39,40,41,42]. Training ML models on a dataset of mixed types of measurements (linear measurements, angles, areas, and indices) has provided correct sex estimation of 95% [5]. In the present study, the highest accuracy of sex estimation (92%) was achieved by SVM on a subset of 19 cranial angles. Most SVM models trained on cranial features have also achieved accuracy of more than 90% [14,15,20]. Indeed, among the different ML algorithms, the SVM models have been the best-performing on datasets of cranial and mandibular measurements [5,43]. They are considered less likely to misclassify a skull because SVM is among the algorithms most resistant to overfitting [14]. In the present study, LR achieved high accuracy, comparable to that of SVM, again on the dataset of cranial angles with AI > 0. LR has performed very well in previous studies on cranial and mandibular measurements, also demonstrating accuracy rates exceeding 90% [5,43]. The NB algorithm delivered an accuracy of 89% on the same subset, a result similar to that of Nikita and Nikitas [18] and notably higher than that obtained by Mota et al. [21]. The highest accuracy of the CN2 rule-induction algorithm was achieved on a smaller subset including only six attributes (85%). The results of the CN2 rules correspond to those of other symbolic algorithms, such as decision trees, that have demonstrated accuracy rates in the range 80-90% [15,16,19]. It could be inferred that all algorithms, except for CN2, have performed better with a larger number of attributes. This suggests that tracing sex differences across more cranial regions enhances the accuracy of sex identification.

**Figure 4:**
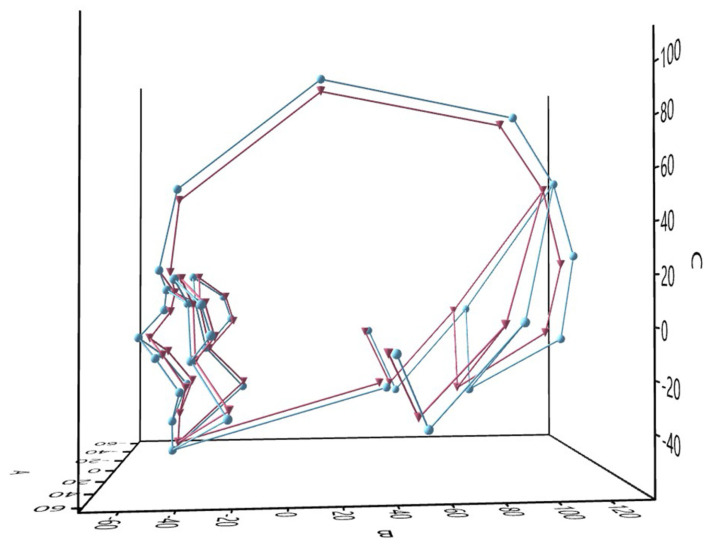
Mean landmark configurations of the male (blue) and female (red) skulls.

The best classification models in this study included almost an equal number of neurocranial and viscerocranial characteristics. There is a discrepancy regarding whether the neurocranium [30] or the viscerocranium [14,42] exhibits more pronounced dimorphic features. Four of the angles were present in all datasets: n-g-m, n-m-FH, fmo-n-fmo, and nm-rhi-nm. All of them have been located at the frontonasoorbital area, including the lower part of the frontal bone (nasion to metopion) and the upper midface. It is well known that the male frontal bone is more inclined and has more prominent glabellar region [6,9,10,11,14,44,45,46,47]. The latter results in a more angled nasofrontal configuration of the male skull compared to the more flattened nasofrontal region of the female one [48]. In fact, the frontal bone contains many dimorphic features, and their metric description has significantly contributed to accurate sex classification [12,45]. The present study also demonstrated that accurate sex identification is closely linked to the involvement of the frontal bone (Figure 4). Aside from the frontal bone, the posterior part of the skull also provides useful sex indicators, such as the nuchal region of the occipital bone and the mastoid processes of the temporal bone. Previous observations have clearly shown that male skulls are characterized by a more prominent and robust occiput [11,14], longer and more projecting mastoids [10,46,49], and a flattening of the lower parietal and lambda region [11]. The sex differences related to these neurocranial features are also evident in the results of the angles included in the present study.

The complex configuration of the facial skeleton undergoes dynamic shape changes during growth and development, especially during the period when sexual dimorphism becomes more pronounced [50,51]. It has been commonly reported that the male midface is more profiled, whereas the female midface is characterized by more subtle angles [14,40,52,53], which corresponds to the more obtuse transversal facial angles (e.g., fmo-n-fmo, zm-ss-zm) observed in the female crania of the studied sample. Concerning the orbital region, female orbits are typically described as relatively larger and more rounded, while male orbits are generally more rectangular in shape [11,40,53,54,55]. In the present study, significant differences were found in the inclination of the orbital height relative to the FH, whereas no such differences were observed in the slope of the orbital breadth axis relative to the FH. The more acute angle of the orbital height in males could be explained by the more protruding supraorbital rim, whereas the superior and inferior orbital borders in females tend to align one over another. This result is consistent with previous observations that the male supraorbital rim projects forward, in contrast to the subtle supraorbital rim in females [48,52]. Additionally, males are often described as having a higher and narrower nasal aperture with more prominent nasal bones, whereas females typically have a wider nasal aperture and flatter nasal bones [11,44,53]. The sex differences described in the nasal region correspond to the results of the present study, which show that the male crania exhibit more acute angles in the nasal ridge (n-mn-rhi) and nasal aperture (nm-rhi-nm, nl-ss-nl) compared to the female crania. Hence, most of the observations described in the former studies are also present by their angular characteristics in the best discriminating subset selected in this study.

The dataset providing the highest accuracy in this study included nine angles measured between a line and a plane and ten angles between two lines. This means that half of these angles were based on the FH. The FH is a reference plane widely used in anthropological and medical practice. The FH has proved to be a stable plane providing small deviations in different measurement trials [56,57]. This makes the FH angles reliable for inclusion in sex estimation models, despite the larger number of landmarks they involve. The advantage of using 3D models is the easy collection of landmark coordinates. This method of collecting metric data facilitates the calculation of various measurements between landmarks. Consequently, landmark coordinates offer greater flexibility in studies, enabling the exploration of a broader and more diverse set of metrics. In the present study, the intraobserver error was assessed by analyzing the acquisition trials of the landmark coordinates. By using standard cranial landmarks with well-defined locations, small deviations were obtained across different trials (less than 1 mm), suggesting high reliability of the derived measurements as well.

Various factors can impact dimensions and spatial relationships between and within bones, with age being one that influences bone morphology. Bones undergo remodeling with age, but the balance between bone resorption and bone formation changes throughout different life stages. With aging, bone loss occurs in specific areas of the facial skeleton, which can significantly contribute to the appearance of the so-called aging face [58,59]. However, not all cranial bones endure resorption. While the midface recedes, the forehead expands, due to the bone deposition in the external wall of the frontal bone (especially in the supraorbital rim) [52]. Age-related changes in the facial skeleton involve an increase in the size of the orbit and pyriform aperture at the expense of the maxillary bone. The latter together with the zygoma undergo anterior and inferior resorption [52,59]. These changes have mainly been studied in relation to cosmetic surgery and their effects on soft tissues (soft-tissue descent and sagging, deepening of folds, lengthening of the lid–cheek junction, drooping of the nose tip, etc. [52]), but they could also reflect on the angular characteristics of the skull and their potential for sex discrimination, which necessitates further examination. 

Cranial morphology varies among different population groups, and population-specificity is an essential part for assessing cranial sexual dimorphism. The present study explored sex differences in cranial angles within a sample of the Bulgarian population. Because of the differences in the strength and expression of sexual dimorphism across various population groups, it is hard to suggest which angles would be the most sexually dimorphic and what classification accuracy they might achieve in other populations. Due to the limited number of studies examining cranial angles as sex indicators, their behavior with respect to population specificity remains unclear. However, the usefulness of cranial angles for sex estimation, as demonstrated by the ML models in this study, could urge their testing on other population groups as well as checking their classification potential by different algorithms and subsets of angles.

## 5. Conclusions

In conclusion, cranial angles are valuable features for sex estimation, and a combination of certain angles can achieve correct sex identification in over 90% of skulls. The most effective models for sex estimation were developed using SVM and LR, based on a dataset of nineteen cranial angles characterizing both neurocranium and viscerocranium. Most of the angles described well-known features for sex discrimination such as glabellar projection, lower forehead inclination, occipital protuberance protrusion, facial profile convexity, etc. Accordingly, the cranial angles demonstrate their usefulness as sex indicators, providing accuracy comparable to that of standard linear measurements. Certainly, combining linear and angular measurements would guarantee greater confidence in the outcome of sex identification in practice.

## Figures and Tables

**Table 1:** Cranial landmarks.

Landmarks		Definition
Midsagittal landmarks	Nasion (n)	The point of intersection between the frontonasal suture and the midsagittal plane.
	Bregma (b)	The point of intersection between the coronal and sagittal sutures.
	Glabella (g)	The most forward-projecting point at the level of the supraorbital ridge in the midsagittal plane.
	Metopion (m)	The point of intersection between the horizontal line connecting the frontal eminences and the midsagittal plane.
	Obelion (ob)	The point of intersection between the line connecting the parietal foramina and the sagittal suture.
	Lambda (l)	The point of intersection between the sagittal and lambdoid sutures.
	Opisthocranion (op)	The most posterior point on the occipital bone in the midsagittal plane; the most distant point from the landmark glabella.
	Inion (i)	The point of intersection between the superior nuchal lines and the midsagittal plane.
	Basion (ba)	The midpoint of the anterior margin of foramen magnum.
	Opisthion (o)	The midpoint of the posterior margin of foramen magnum.
	Rhinion (rhi)	The point of intersection between the internasal suture and the margin of the piriform aperture.
	Subspinale (ss)	The most posterior point on the intermaxillary suture, immediately below the anterior nasal spine.
	Prosthion (pr)	The most anterior point on the upper alveolar process in the midsagittal plane.
	Midnasale (mn)	The deepest midline point on the nasal bones.
Bilateral landmarks	Frontomalare-orbitale (fmo)	The point of intersection between the zygomaticofrontal suture and the lateral orbital margin.
	Asterion (ast)	The point of intersection between the lambdoid, parietomastoid, and occipitomastoid sutures.
	Mastoidale (ms)	The most inferior point on the tip of the mastoid process.
	Maxillofrontale (mf)	The point of intersection between the frontomaxillary suture and the medial orbital margin.
	Ektokonchion (ek)	The point of intersection between the line beginning from the landmark maxillofrontale and crossing the orbit parallel to the superior orbital margin and the lateral orbital margin.
	Supraorbitale (so)	The most superior point on the superior orbital margin.
	Zygoorbitale (zo)	The point of intersection between the zygomaxillary suture and the inferior orbital margin.
	Nasolaterale (nl)	The most lateral point on the margin of the piriform aperture.
	Zygomaxillare (zm)	The most inferior point on the zygomaticomaxillary suture.
	Nasomaxillare (nm)	The point of intersection between the nasomaxillary suture and the margin of the piriform aperture.
	Porion (po)	The most superior point on the margin of the external auditory meatus.
	Orbitale ^#^ (or)	The most inferior point on the inferior margin of the orbit.

^#^ acquired only on the left side.

**Table 2:** Cranial angles.

Angles		Description
Angles between a line and a plane	Frontal slope angle (n-b-FH)	The angle between the line nasion-bregma and the FH *.
	Frontal profile angle (n-m-FH)	The angle between the line nasion-metopion and the FH.
	Parietal slope angle(ob-l-FH)	The angle between the line obelion-lambda and the FH.
	Lambda-opisthocranion angle (l-op-FH)	The angle between the line lambda-opisthocranion and the FH.
	Lambda-inion angle (l-i-FH)	The angle between the line lambda-inion and the FH.
	Opisthocranion-inion angle (op-i-FH)	The angle between the line opisthocranion-inion and the FH.
	Foramen magnum tilt angle (ba-o-FH)	The angle between the line basion-opisthion and the FH.
	Calottebasis angle (n-i-FH)	The angle between the line nasion-inion and the FH.
	Glabella-lambda angle (g-l-FH)	The angle between the line glabella-lambda and the FH.
	Glabella-inion angle (g-i-FH)	The angle between the line glabella-inion and the FH.
	Facial profile angle (n-pr-FH)	The angle between the line nasion-prosthion and the FH.
	Nasal profile angle (n-ss-FH)	The angle between the line nasion-subspinale and the FH.
	Alveolar profile angle (ss-pr-FH)	The angle between the line subspinale-prosthion and the FH.
	Nasal bones angle (n-rhi-FH)	The angle between the line nasion-rhinion and the FH.
	Zygomaticomaxillary suture tilt angle ^b^ (zm-zo-FH)	The angle between the line zygomaxillare-zygoorbitale and the FH.
	Sagittal tilt of the orbit entrance ^b^ (so-zo-FH)	The angle between the line supraorbitale-zygoorbitale and the FH.
	Horizontal tilt of the orbit entrance ^b^ (ek-mf-FH)	The angle between the line ektokonchion-maxillofrontale and the FH.
Angles between two lines	Frontal curvature angle (n-m-b)	The angle constructed between the landmarks nasion, metopion, and bregma with vertex at the metopion.
	Glabellar angle (n-g-m)	The angle constructed between the landmarks nasion, glabella, and metopion with vertex at the glabella.
	Upper occipital angle (l-op-i)	The angle constructed between the landmarks lambda, opisthocranion, and inion with vertex at the opisthocranion.
	Prosthion-glabella-lambda angle (pr-g-l)	The angle constructed between the lines prosthion-glabella and glabella-lambda.
	Facial triangle angle (n-pr-ba)	The angle at prosthion in the facial triangle nasion-prosthion-basion.
	Nasomalar angle (fmo-n-fmo)	The angle constructed between the right frontomalare orbitale, nasion, and the left frontomalare orbitale, with vertex at the nasion.
	Zygomaxillary angle (zm-ss-zm)	The angle constructed between the right zygomaxillare, subspinale, and the left zygomaxillare, with vertex at the subspinale
	Nasofrontal angle (g-n-rhi)	The angle constructed between the landmarks glabella, nasion, and rhinion with vertex at the nasion.
	Nasal bone projection angle towards upper facial height (rhi-n-pr)	The angle constructed between the lines nasion-rhinion and nasion-prosthion.
	Nasal bone projection angle towards nasal height (rhi-n-ss)	The angle constructed between the lines nasion-rhinion and nasion-subspinale.
	Nasal bone curvature angle (n-mn-rhi)	The angle constructed between the landmarks nasion, midnasale, and rhinion with vertex at the midnasale.
	Nasomaxillare-rhinion angle (nm-rhi-nm)	The angle constructed between the right nasomaxillare, rhinion, and the left nasomaxillare, with vertex at the rhinion.
	Maxillofrontal angle (mf-n-mf)	The angle constructed between the right maxillofrontale, nasion, and the left maxillofrontale, with vertex at the nasion.
	Nasolateral angle (nl-ss-nl)	The angle constructed between the right nasolaterale, subspinale, and the left nasolaterale, with vertex at the subspinale.
	Mastoid angle ^b^ (po-ms-ast)	The angle constructed between the landmarks porion, mastoidale, and asterion, with vertex at the mastoidale.

* FH—Frankfurt horizontal plane; ^b^—bilateral.

**Table 3:** Descriptive and test statistics of the cranial angles.

Angles	Males					Females					Sex Differences *p*-Value
	n	Mean	SD	Min	Max	n	Mean	SD	Min	Max	
n-b-FH	154	49.77	3.00	43.50	57.12	180	50.24	2.77	39.18	57.44	0.113 ^t^
n-m-FH	154	79.62	4.18	68.98	89.47	180	82.89	3.84	69.49	89.54	<0.001 *^t^
n-m-b	154	133.76	4.42	117.81	143.91	180	130.45	4.10	120.62	142.62	<0.001 *^t^
n-g-m	154	146.67	8.05	130.00	165.17	180	160.15	6.58	146.97	174.77	<0.001 *^U^
ob-l-FH	154	62.23	5.18	47.91	80.83	180	59.71	5.09	41.76	74.67	<0.001 *^t^
l-op-FH	154	77.95	4.07	67.11	89.13	180	79.33	4.07	67.19	89.93	0.002 *^t^
l-i-FH	154	85.28	2.86	73.78	89.82	180	84.77	3.16	74.62	89.55	0.207 ^U^
op-i-FH	154	76.72	5.66	59.04	88.45	180	72.50	6.18	59.01	89.53	<0.001 *^t^
l-op-i	154	155.37	6.03	141.12	169.07	180	152.36	5.99	137.73	167.22	<0.001 *^t^
ba-o-FH	154	6.66	4.57	0.04	30.73	180	8.17	4.61	0.01	21.50	0.002 *^U^
n-i-FH	154	11.60	2.82	4.63	17.99	180	10.46	3.09	1.68	21.41	<0.001 *^t^
g-l-FH	154	8.05	2.92	0.25	15.13	180	8.36	3.10	0.59	17.54	0.347 ^t^
g-i-FH	154	14.16	2.75	7.22	20.35	180	13.38	3.06	5.14	23.93	0.014 *^t^
n-pr-FH	136	86.49	2.38	74.32	89.77	166	86.16	2.42	77.19	89.67	0.225 ^U^
pr-g-l	136	98.45	3.84	86.50	108.61	166	99.92	3.61	90.76	108.94	0.001 *^U^
n-pr-ba	136	76.21	3.49	64.89	86.17	166	75.31	2.88	67.71	82.00	0.015 *^t^
n-ss-FH	154	86.55	2.22	74.61	89.95	180	86.61	2.14	79.59	89.63	0.691 ^U^
ss-pr-FH	136	81.45	5.90	39.27	89.37	166	80.54	5.70	57.84	89.04	0.101 ^U^
fmo-n-fmo	154	133.54	5.35	121.13	149.32	180	137.21	4.13	128.43	149.04	<0.001 *^U^
zm-ss-zm	154	115.88	5.30	101.46	131.04	180	118.30	4.85	105.00	133.01	<0.001 *^t^
g-n-rhi	154	129.32	8.10	107.22	149.65	180	137.99	6.34	119.77	153.61	<0.001 *^U^
zm-zo-FH (R)	154	43.80	5.04	25.10	58.20	180	42.18	4.63	28.58	59.29	0.002 *^t^
zm-zo-FH (L)	154	43.92	4.52	32.11	55.64	180	42.23	4.24	30.19	53.84	<0.001 *^t^
so-zo-FH (R)	154	79.30	3.72	63.28	87.15	180	80.88	3.51	70.44	89.29	<0.001 *^U^
so-zo-FH (L)	154	80.25	3.78	70.90	89.14	180	81.52	3.24	67.69	88.74	0.002 *^U^
ek-mf-FH (R)	154	15.24	3.24	7.71	23.98	180	15.45	3.10	7.74	24.60	0.558 ^t^
ek-mf-FH (L)	154	16.51	3.39	7.87	23.95	180	16.40	3.36	7.42	26.31	0.771 ^t^
rhi-n-ss	154	32.42	4.67	20.68	47.95	180	31.30	4.58	21.01	45.39	0.032 *^U^
rhi-n-pr	136	30.09	5.23	18.65	48.88	166	30.11	4.78	19.75	45.20	0.002 *^U^
n-mn-rhi	154	150.28	8.74	120.12	174.04	180	154.79	7.60	130.97	175.47	<0.001 *^t^
nm-rhi-nm	154	74.53	9.48	48.49	108.32	180	80.91	10.08	54.07	104.78	<0.001 *^t^
mf-n-mf	154	87.53	9.01	68.25	116.56	180	90.23	9.62	64.53	117.97	0.009 *^t^
nl-ss-nl	154	80.20	7.23	49.34	101.81	180	83.87	7.32	64.28	106.35	<0.001 *^U^
n-rhi-FH	154	55.82	5.78	36.23	71.21	180	56.79	5.39	41.53	70.19	0.112 ^t^
po-ms-ast (R)	154	65.40	6.42	50.73	81.75	180	68.02	6.48	50.78	89.83	<0.001 *^t^
po-ms-ast (L)	154	65.45	5.99	51.78	86.72	180	67.83	5.98	56.32	84.62	<0.001 *^U^

R—right; L—left; *—*p* < 0.05; t—*t*-test; U—U-test.

**Table 4:** Attribute importance (AI) of the cranial angles.

AI	Attributes	Number
>0	n-m-FH *, n-g-m, ob-l-FH, l-op-FH, op-i-FH, l-op-i, ba-o-FH, n-i-FH, po-ms-ast (left), n-pr-ba, fmo-n-fmo, zm-ss-zm, g-n-rhi, zm-zo-FH (left), so-zo-FH (right), so-zo-FH (left), n-mn-rhi, nm-rhi-nm, nl-ss-nl	19
≥0.1	n-m-FH, n-g-m, ob-l-FH, op-i-FH, po-ms-ast (left), fmo-n-fmo, zm-ss-zm, g-n-rhi, zm-zo-FH (left), so-zo-FH (right), so-zo-FH (left), n-mn-rhi, nm-rhi-nm, nl-ss-nl	14
≥0.3	n-m-FH, n-g-m, ob-l-FH, op-i-FH, po-ms-ast (left), fmo-n-fmo, g-n-rhi, zm-zo-FH (left), so-zo-FH (right), n-mn-rhi, nm-rhi-nm, nl-ss-nl,	12
≥0.4	n-m-FH, n-g-m, ob-l-FH, op-i-FH, fmo-n-fmo, g-n-rhi, n-mn-rhi, nm-rhi-nm, nl-ss-nl	9
≥0.6	n-m-FH, n-g-m, op-i-FH, fmo-n-fmo, g-n-rhi, nm-rhi-nm, nl-ss-nl	7
≥0.9	n-m-FH, n-g-m, op-i-FH, fmo-n-fmo, g-n-rhi, nm-rhi-nm	6
=1	n-m-FH, n-g-m, fmo-n-fmo, nm-rhi-nm	4

* FH—Frankfurt horizontal plane.

## Data Availability

The data presented in the study are available on request from the corresponding author.

## Supplementary Materials

The following supporting information can be downloaded at: https://www.mdpi.com/article/10.3390/biology13100780/s1, Supplementary File S1: Visualization of the cranial angles; Supplementary File S2: Accuracy of the ML models on the separate attribute datasets; Table S1: Intraobserver measurement error of the landmarks (in mm).
