# Supplementary material for: Enhancing Sex Estimation Accuracy with Cranial Angle Measurements and Machine Learning
Source: Biology (Basel). 2024 Sep 29;13(10):780. doi: 10.3390/biology13100780 (PMC11504716; doi:10.3390/biology13100780)

Supplementary File S1. Visualization of the cranial angles.

Each cranial angle used in the study is visualized in the free software Geomagic Verify Viewer 2015.2.0 (3D Systems, Inc.). The software was not used for measurements in the present study, but it allows measuring angles between three points, between two lines and between a line and a plane.

n-b-FH

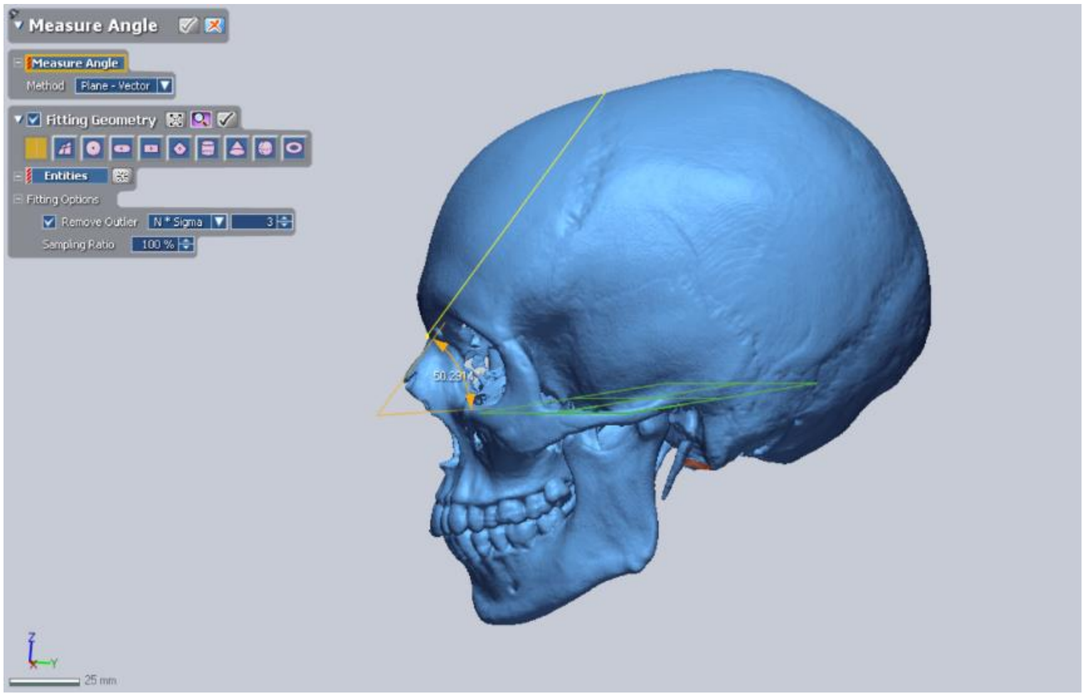

n-m-FH

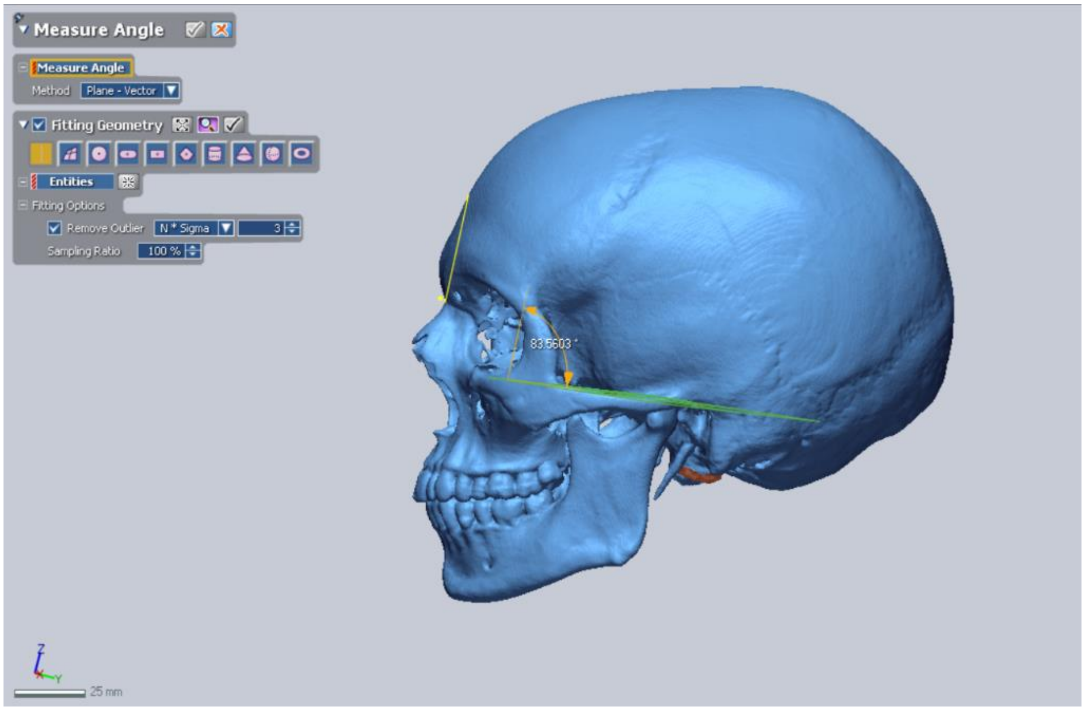

n-m-b

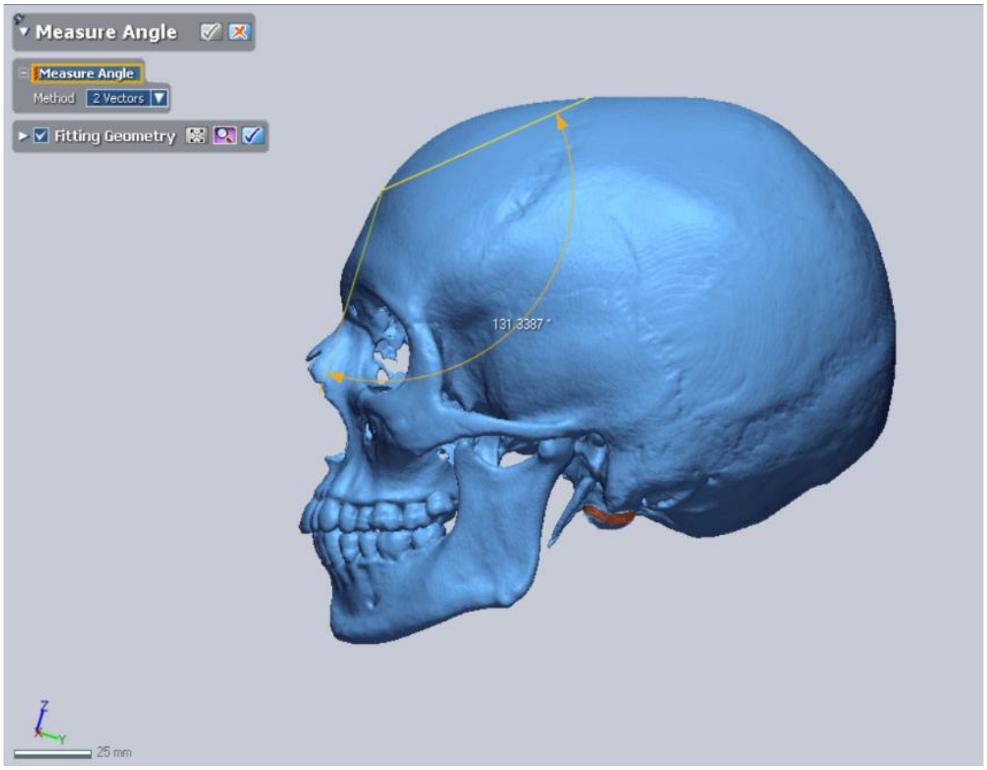
Measure Angle
Measure Angle
Method
2 Vectors
Fitting Geometry
131.3387 °
25 mm

n-g-m

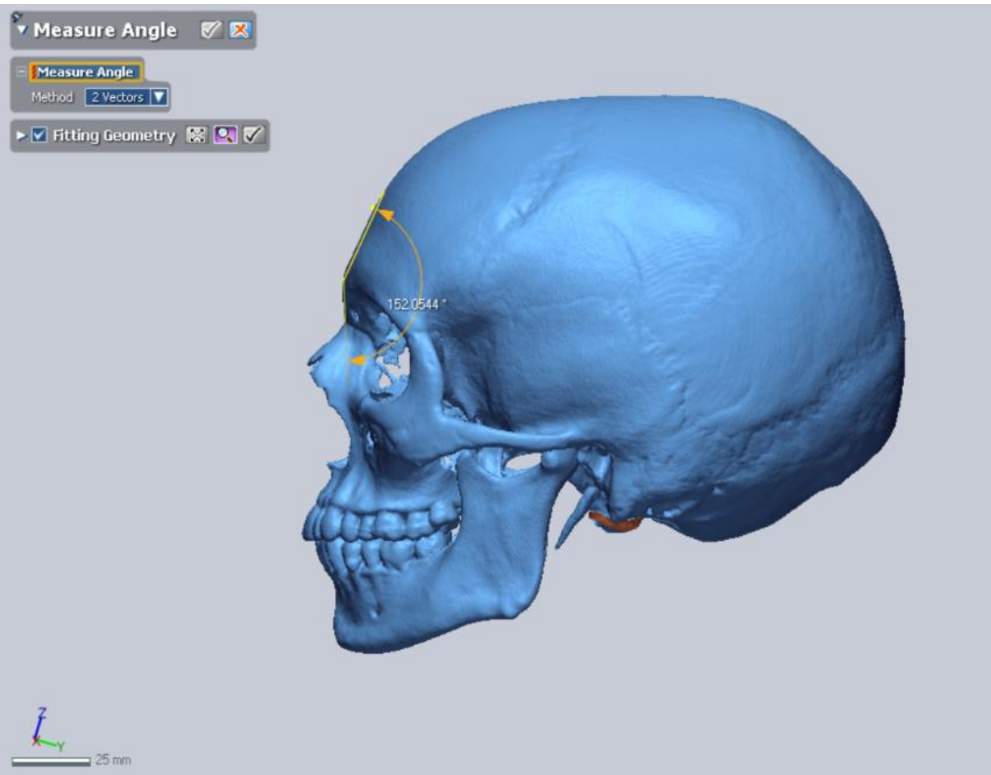
Measure Angle
Measure Angle
Method
2 Vectors
Fitting Geometry
152.0544 °
25 mm

ob-l-FH

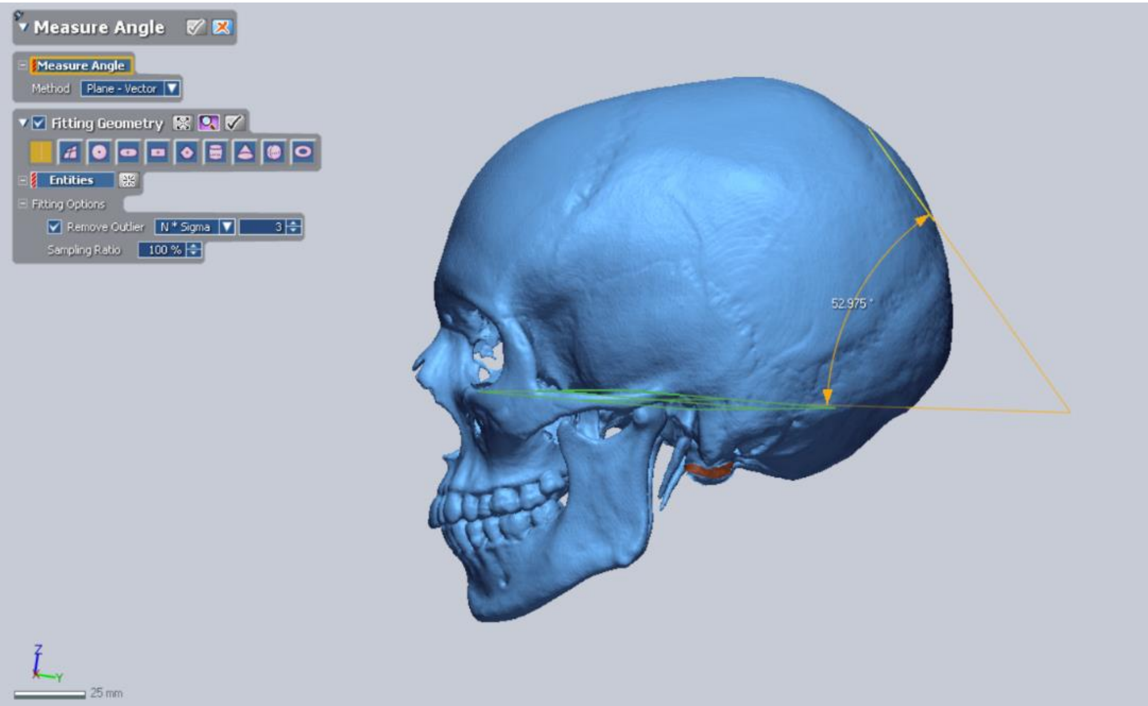
Measure Angle
Measure Angle
Method
Plane - Vector
Fitting Geometry
Entities
Fitting Options
Remove Outlier
N * Sigma
3
Sampling Ratio
100 %
52.975 °
25 mm

l-op-FH

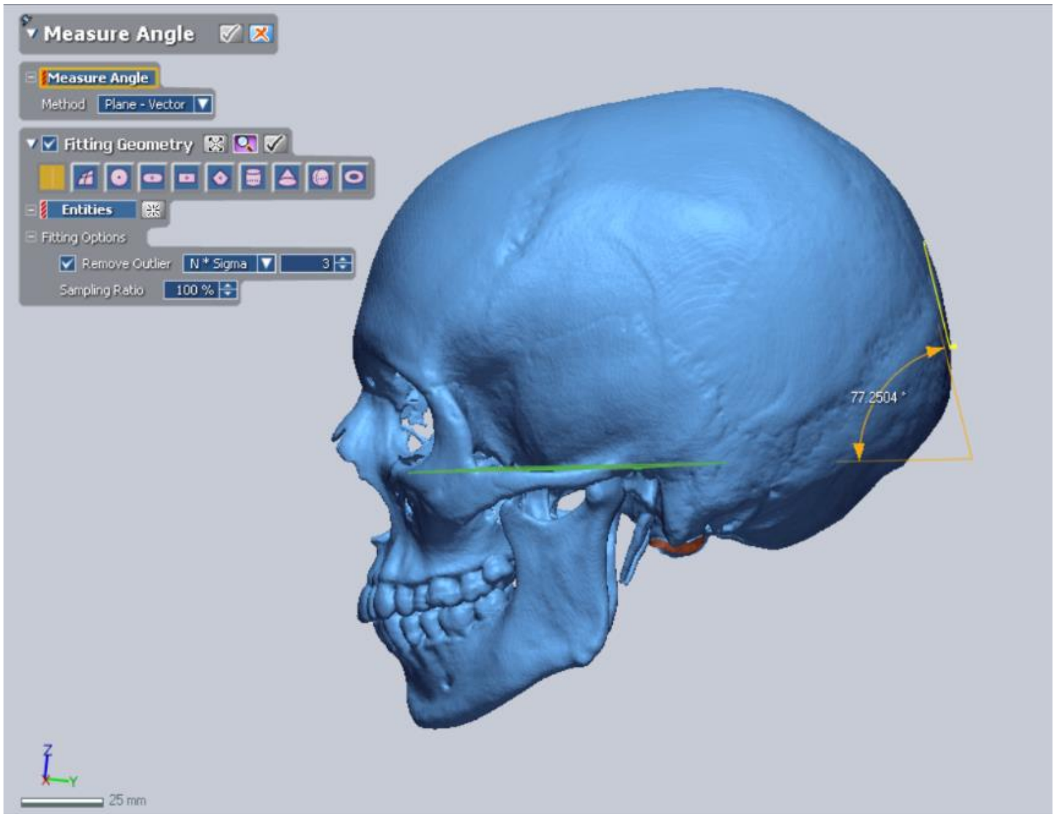
Measure Angle
Measure Angle
Method
Plane - Vector
Fitting Geometry
Entities
Fitting Options
Remove Outlier
N * Sigma
3
Sampling Ratio
100 %
77.2504 °
25 mm

l-i-FH

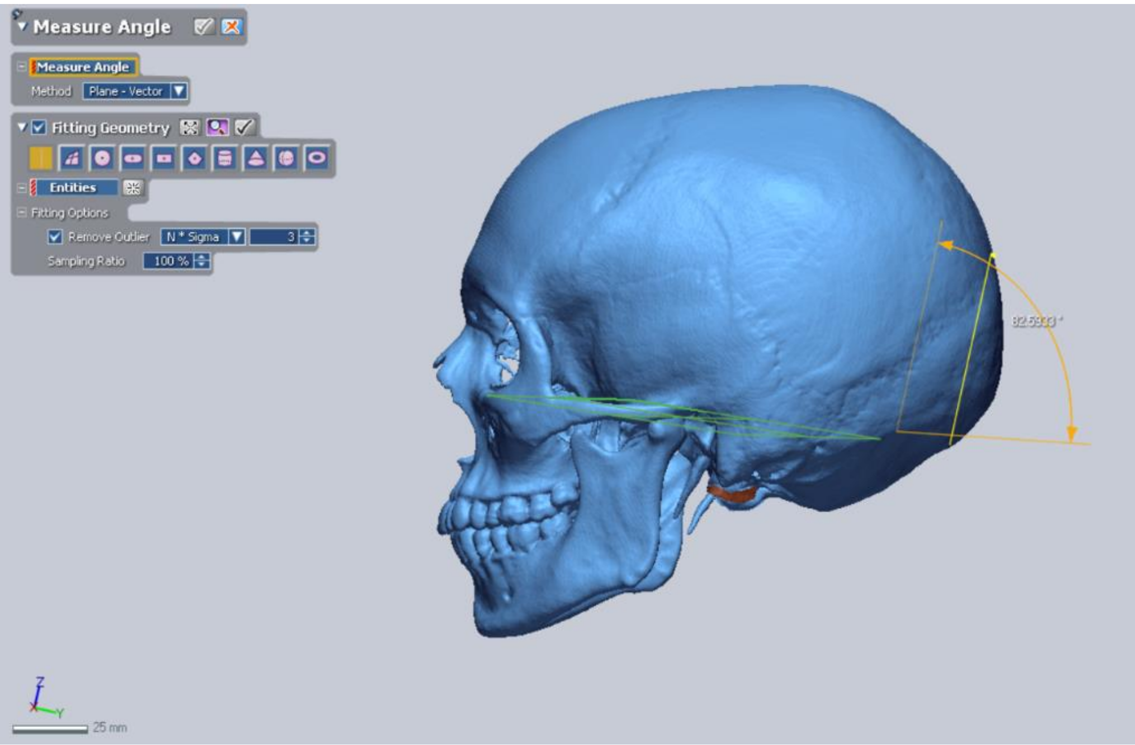
Measure Angle
Measure Angle
Method
Plane - Vector
Fitting Geometry
Entities
Fitting Options
Remove Outlier
N * Sigma
3
Sampling Ratio
100 %
82.5933 °
25 mm

op-i-FH

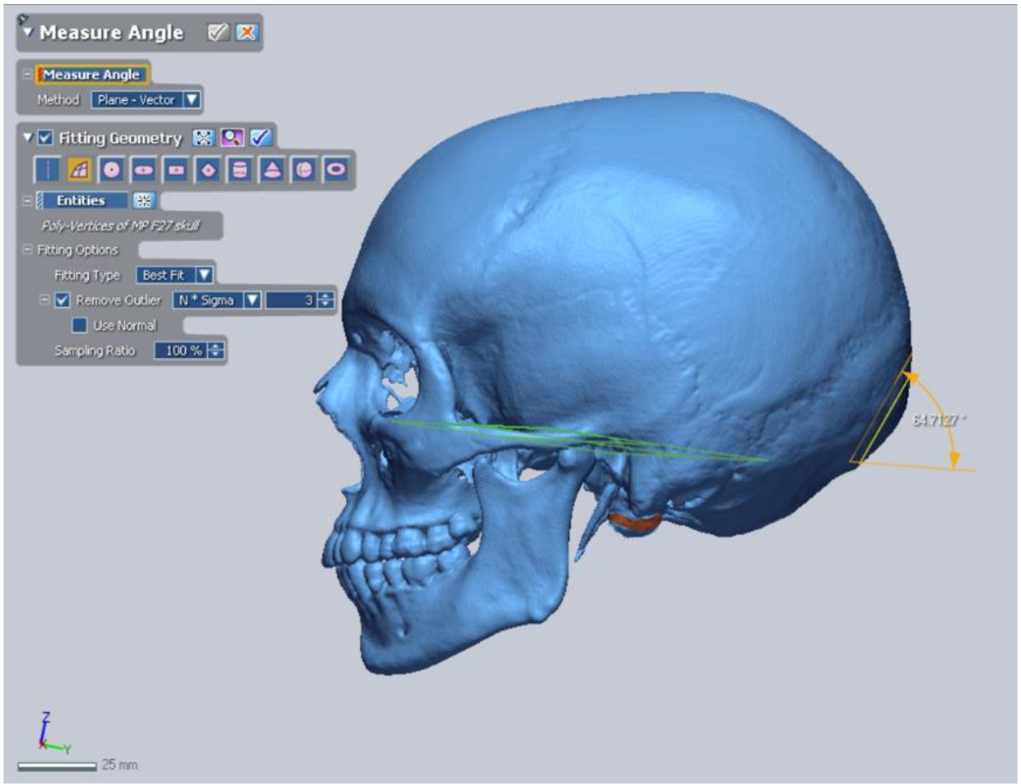
Measure Angle
Measure Angle
Method
Plane - Vector
Fitting Geometry
Entities
Poly-Vertices of MP F27 skull
Fitting Options
Fitting Type
Best Fit
Remove Outlier
N * Sigma
3
Use Normal
Sampling Ratio
100 %
64.7127 °
25 mm

l-op-i

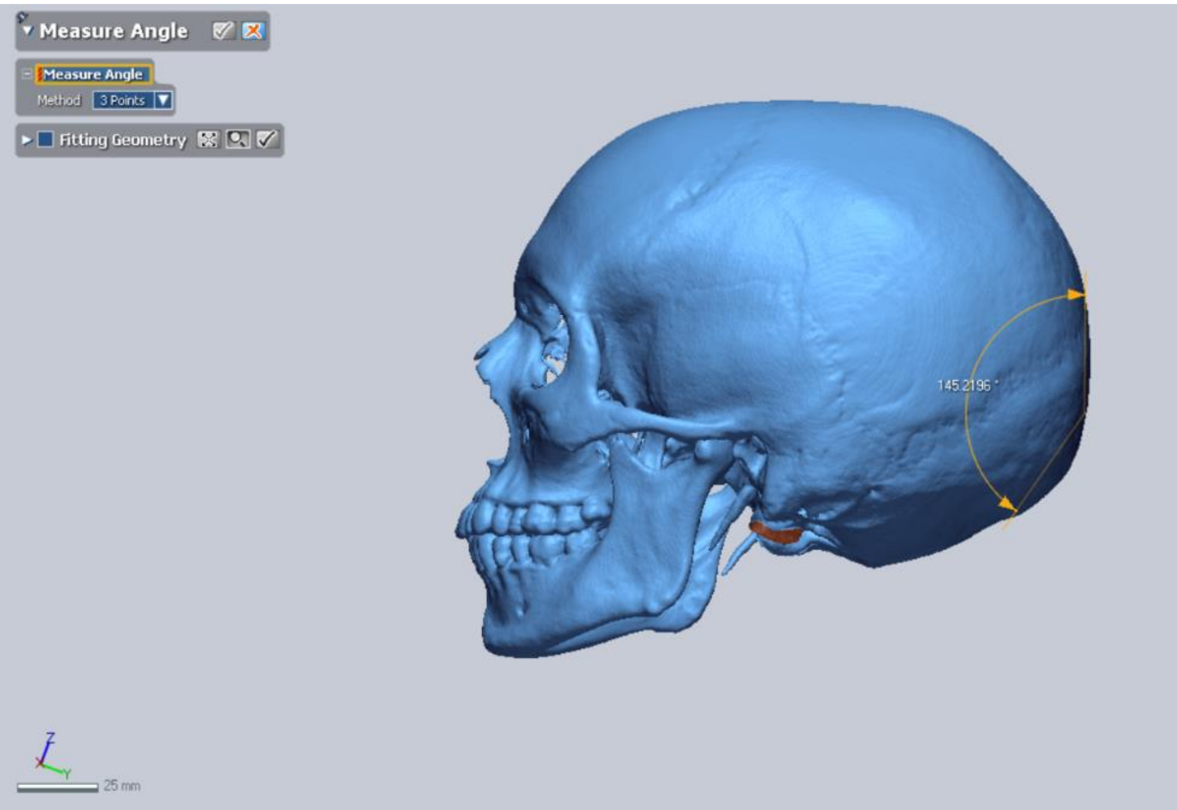
Measure Angle
Measure Angle
Method 3 Points
Fitting Geometry
145.2196 °
25 mm

ba-o-FH

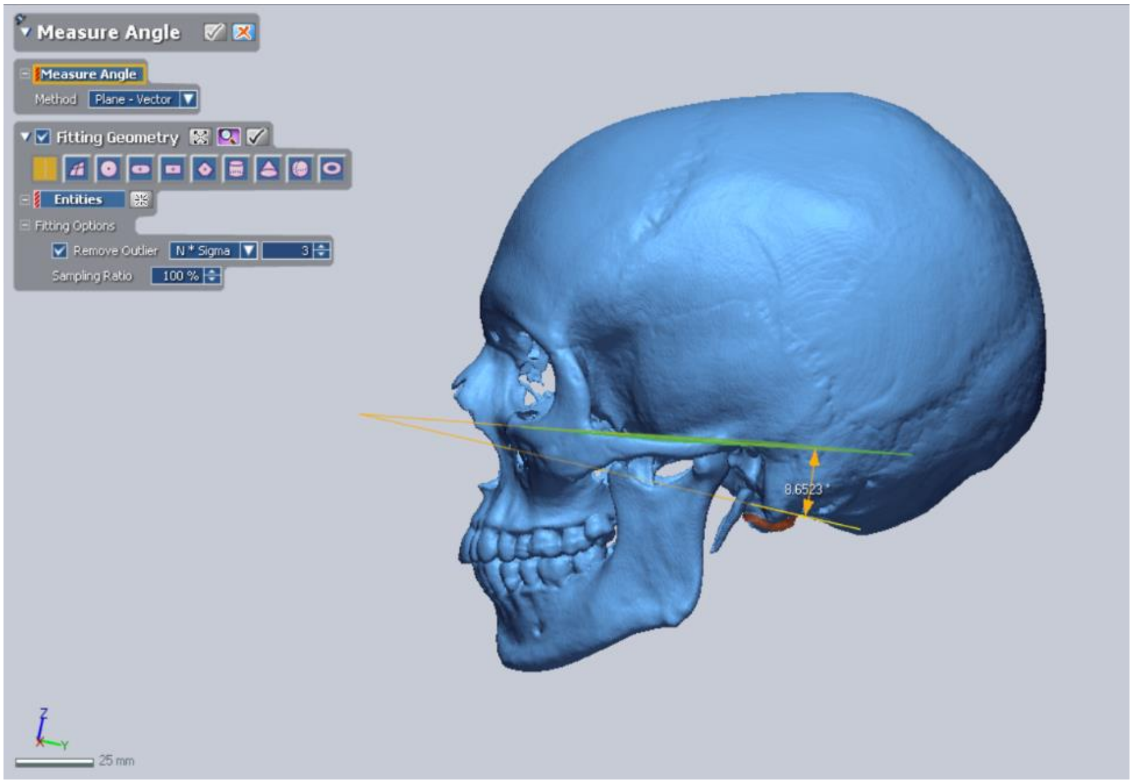
Measure Angle
Measure Angle
Method Plane - Vector
Fitting Geometry
Entities
Fitting Options
Remove Outlier N * Sigma 3
Sampling Ratio 100 %
8.6523 °
25 mm

n-i-FH

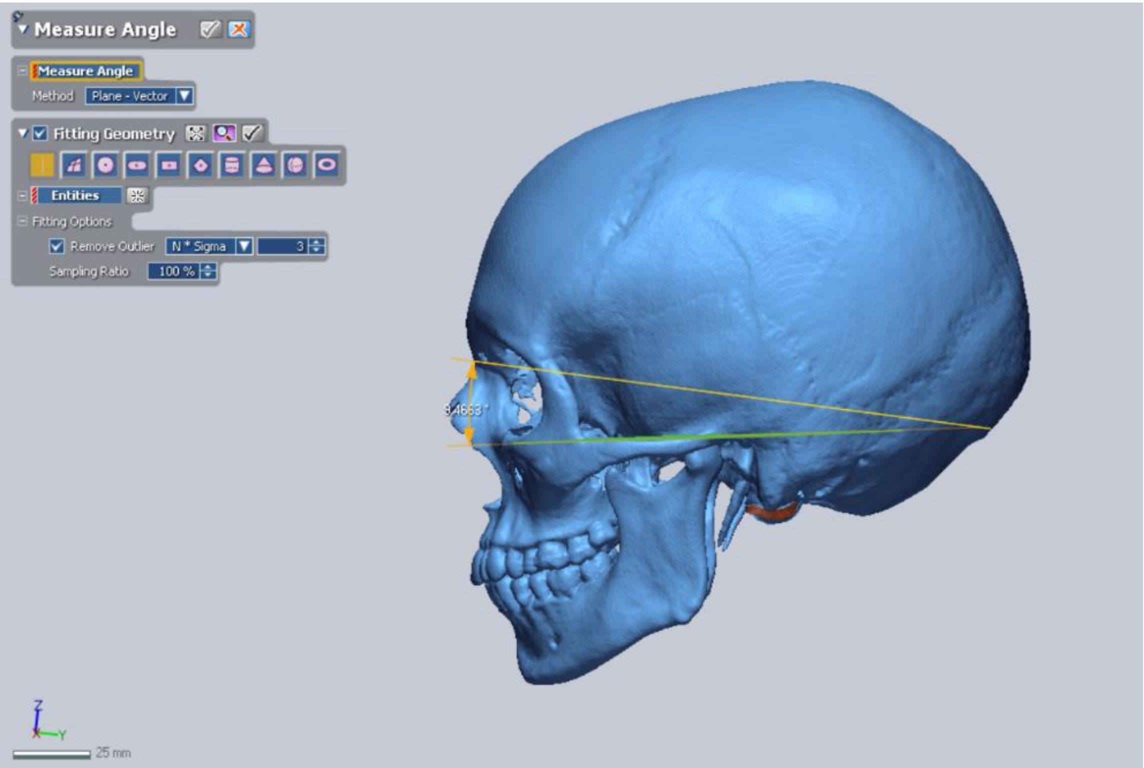
Measure Angle
Measure Angle
Method
Plane - Vector
Fitting Geometry
Entities
Fitting Options
Remove Outlier
N * Sigma
3
Sampling Ratio
100 %
25 mm

g-l-FH

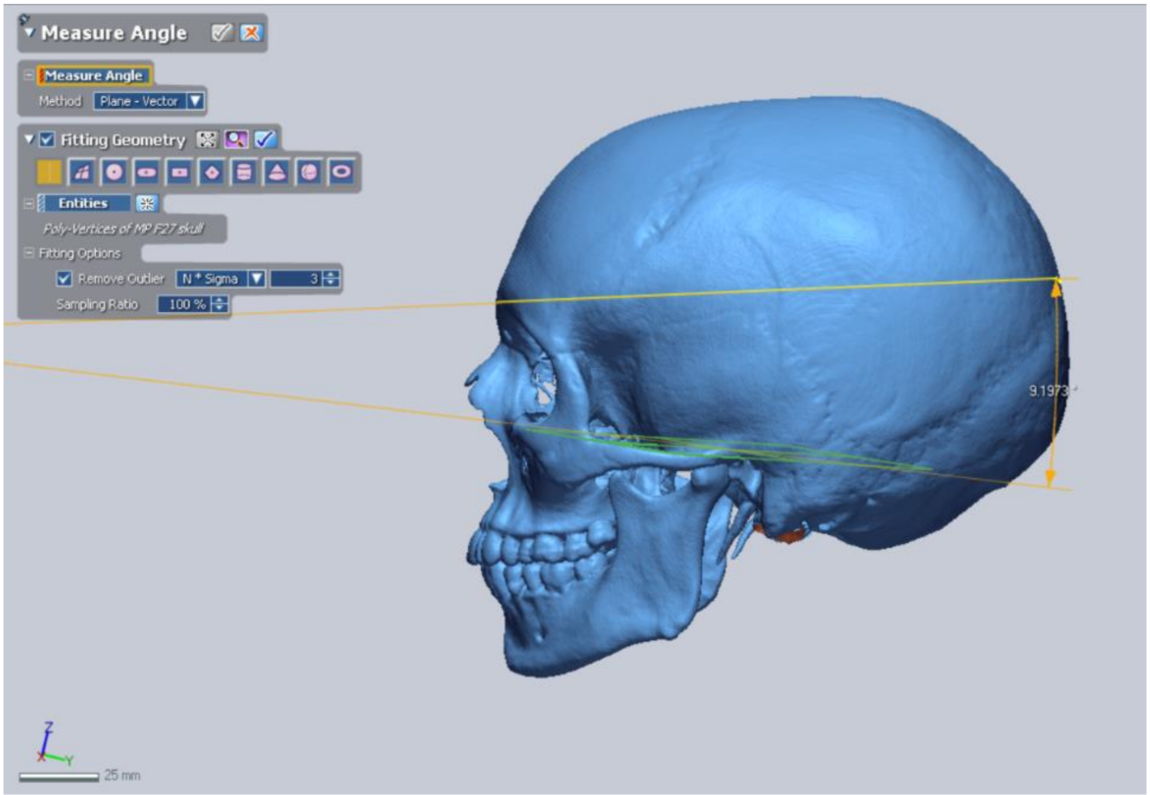
Measure Angle
Measure Angle
Method
Plane - Vector
Fitting Geometry
Entities
Poly-Vertices of MP F27 skull
Fitting Options
Remove Outlier
N * Sigma
3
Sampling Ratio
100 %
9.1973 °
25 mm

g-i-FH

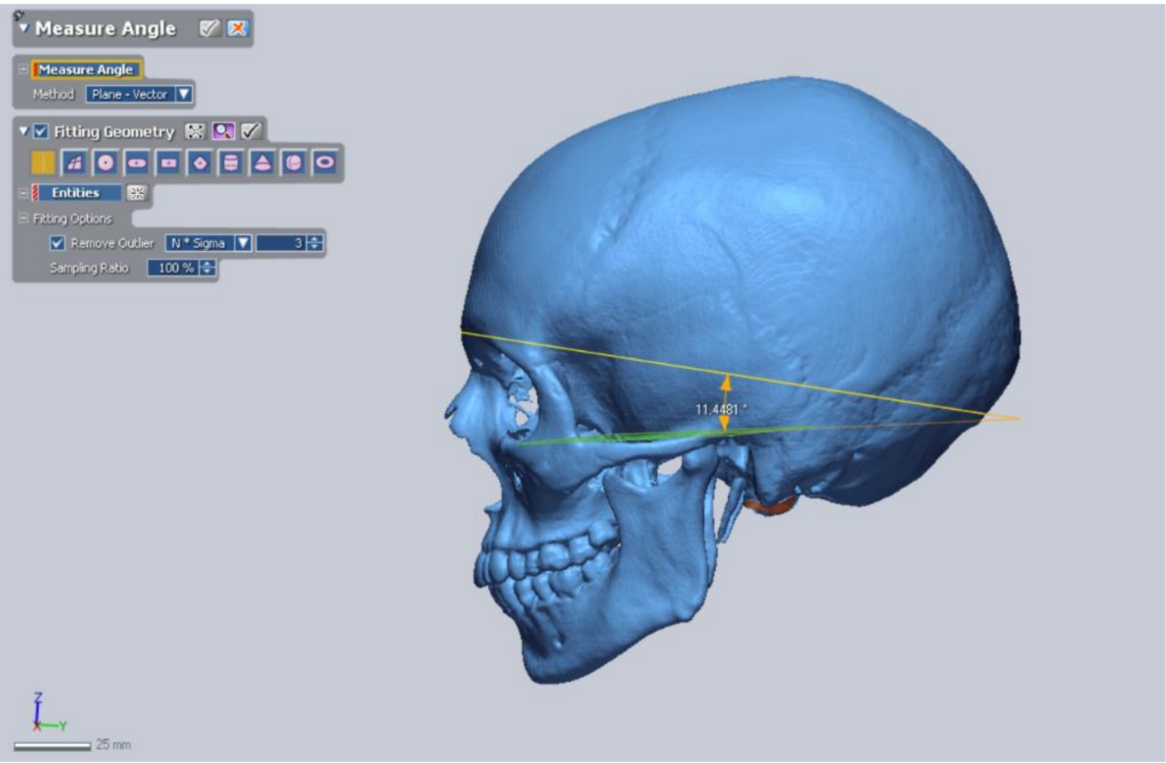
Measure Angle
Measure Angle
Method
Plane - Vector
Fitting Geometry
Entities
Fitting Options
Remove Outlier
N * Sigma
3
Sampling Ratio
100 %
11.4481 °
25 mm

n-pr-FH

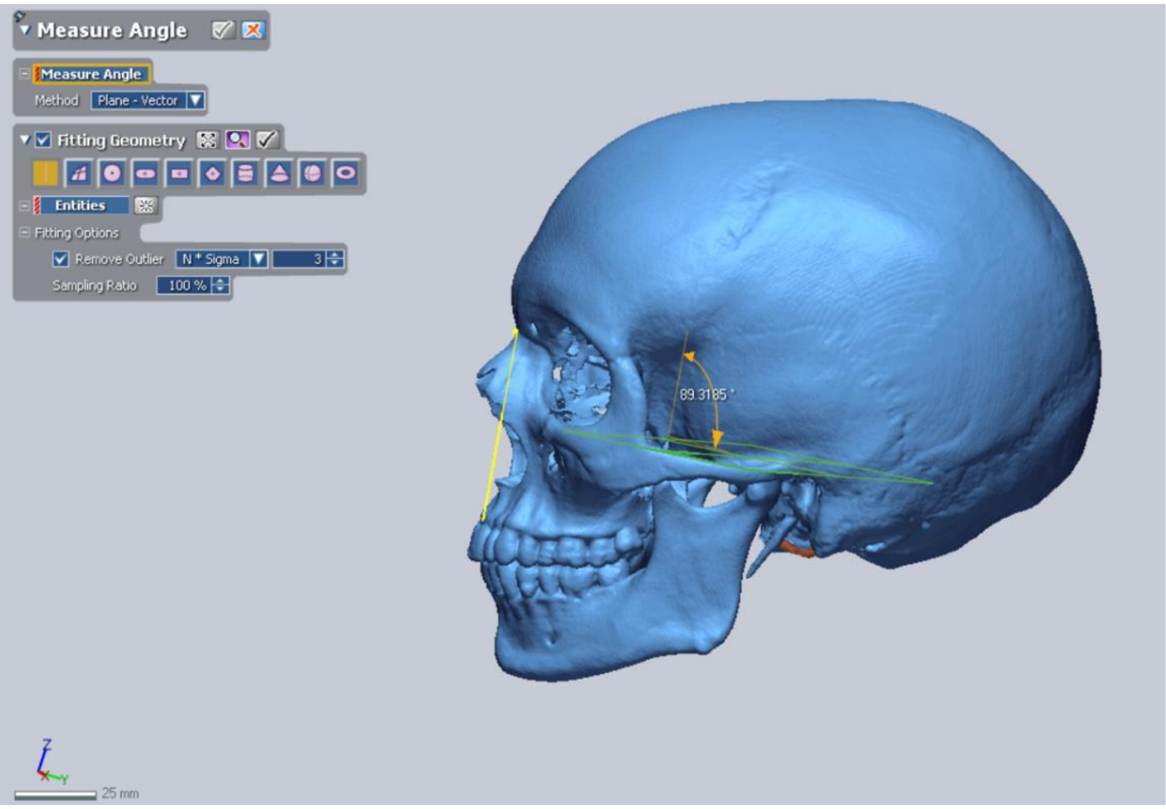
Measure Angle
Measure Angle
Method
Plane - Vector
Fitting Geometry
Entities
Fitting Options
Remove Outlier
N * Sigma
3
Sampling Ratio
100 %
89.3185 °
25 mm

pr-g-l

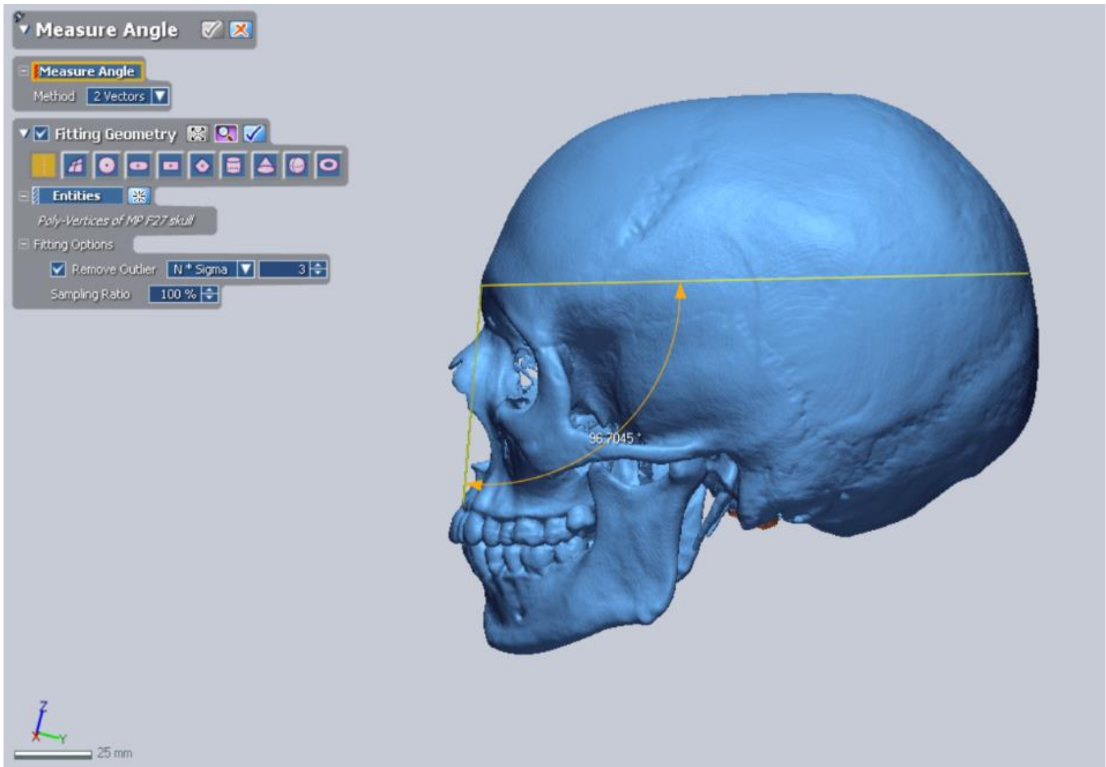
Measure Angle
Measure Angle
Method
2 Vectors
Fitting Geometry
Entities
Poly-Vertices of MP F27 skull
Fitting Options
Remove Outlier
N * Sigma
3
Sampling Ratio
100 %
96.7045 °
25 mm

n-pr-ba

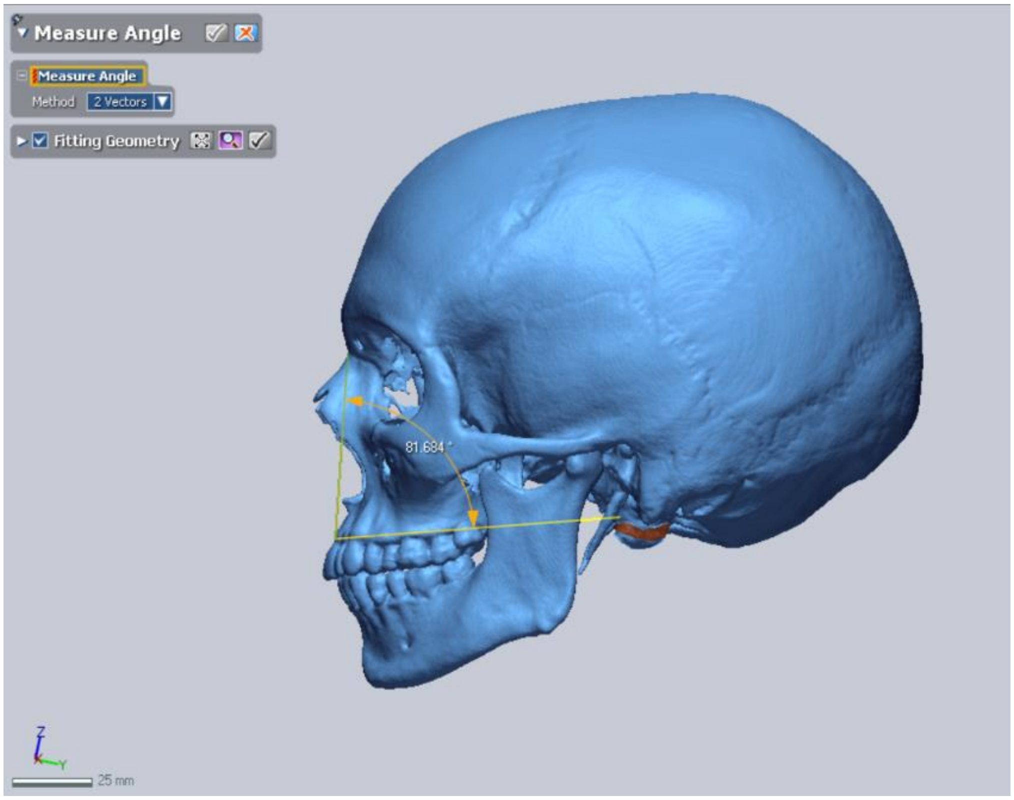
Measure Angle
Measure Angle
Method
2 Vectors
Fitting Geometry
81.684 °
25 mm

n-ss-FH

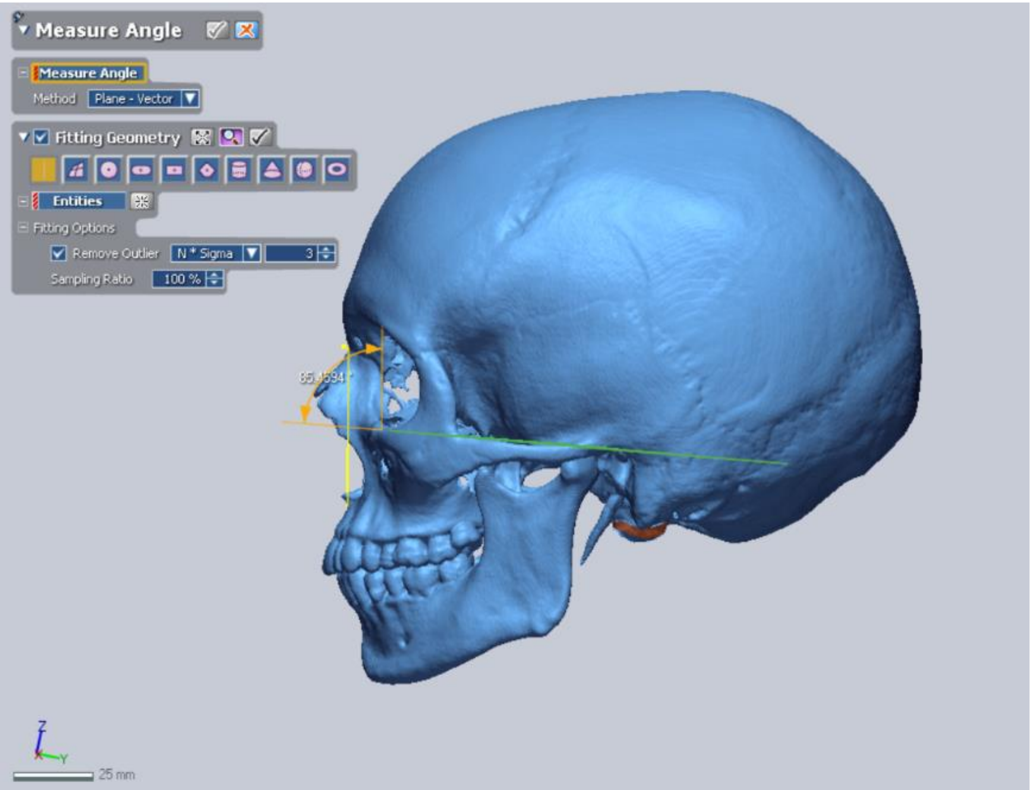

ss-pr-FH

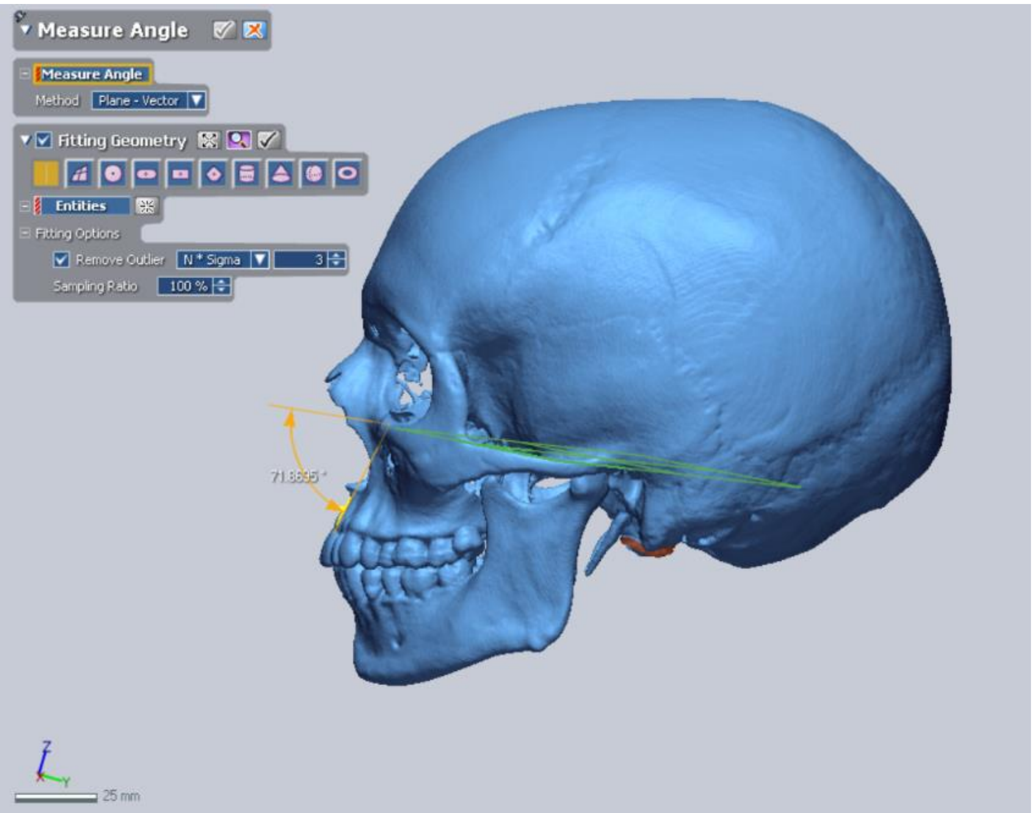

fmo-n-fmo

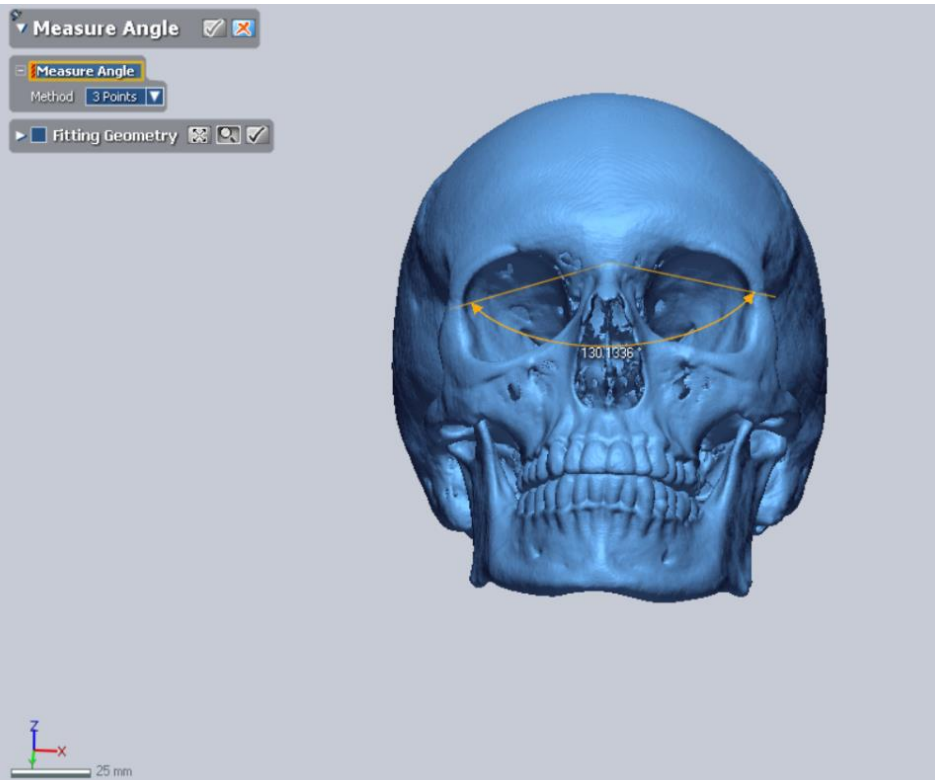

zm-ss-zm

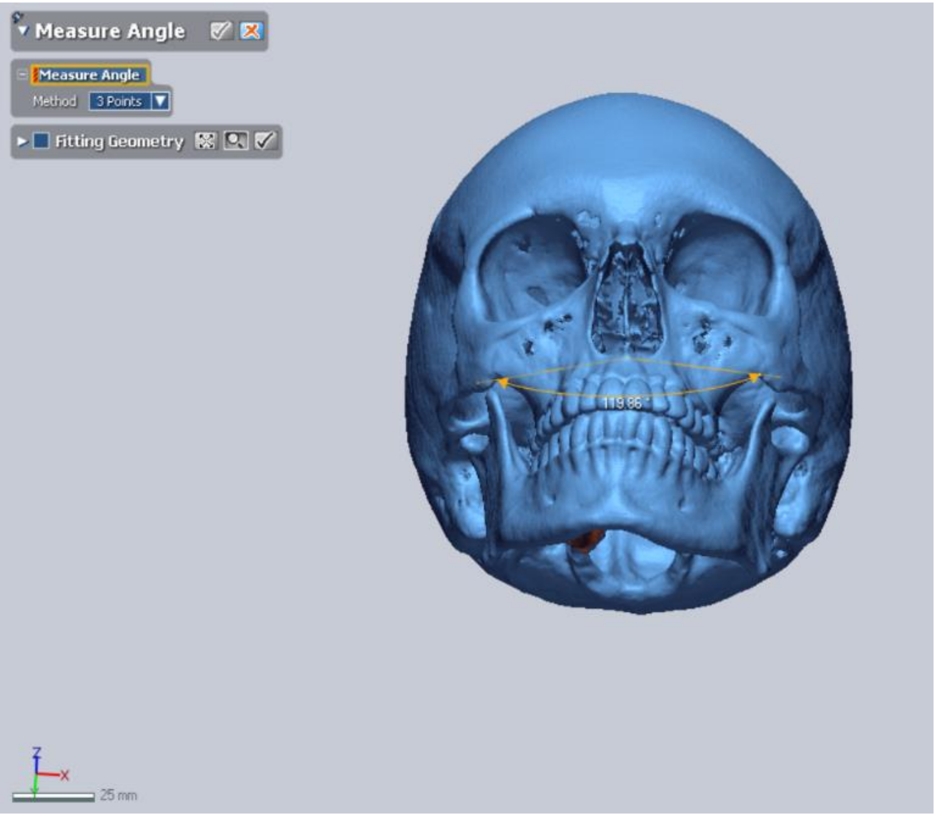

g-n-rhi

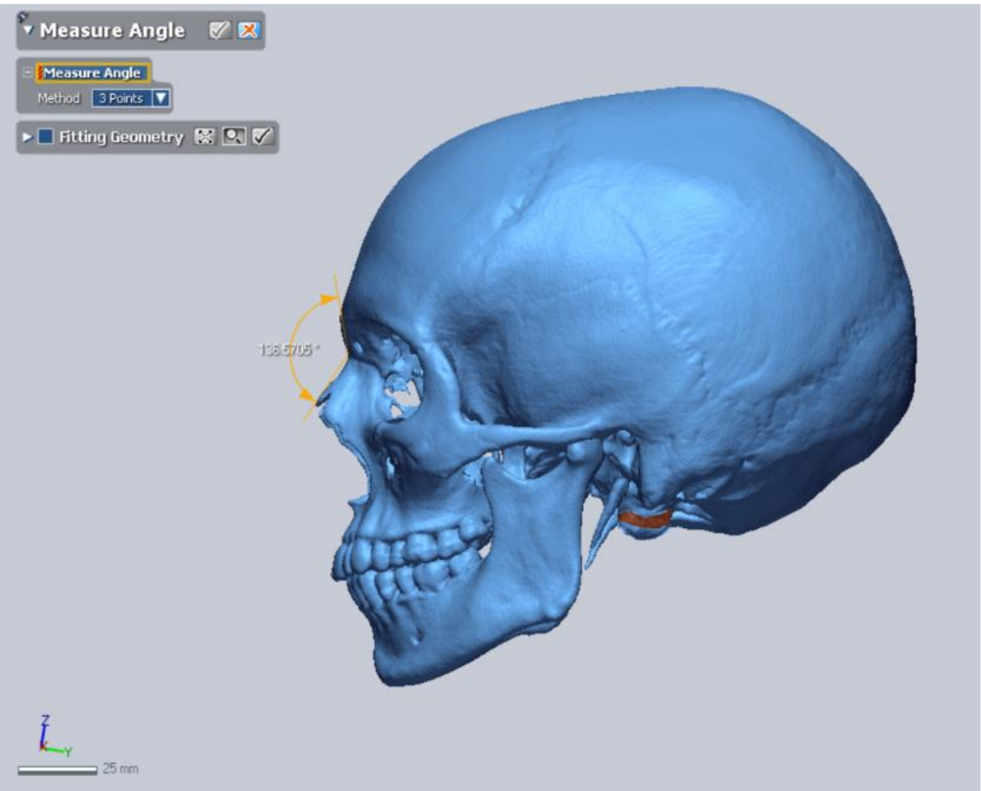

zm-zo-FH

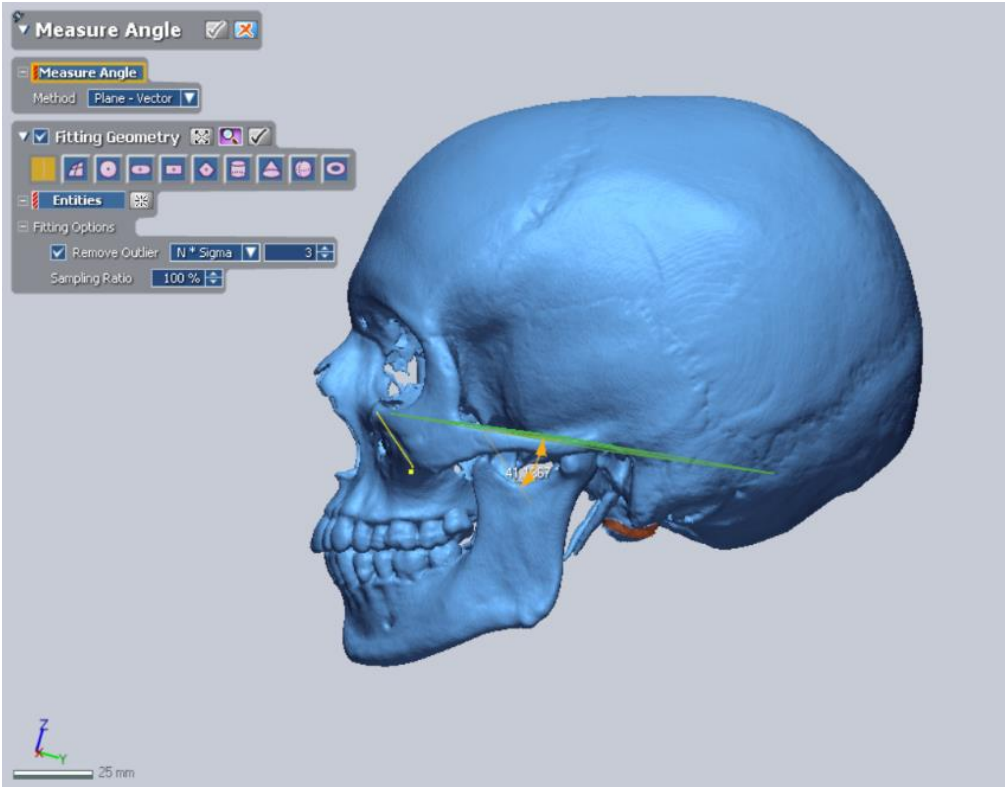

so-zo-FH

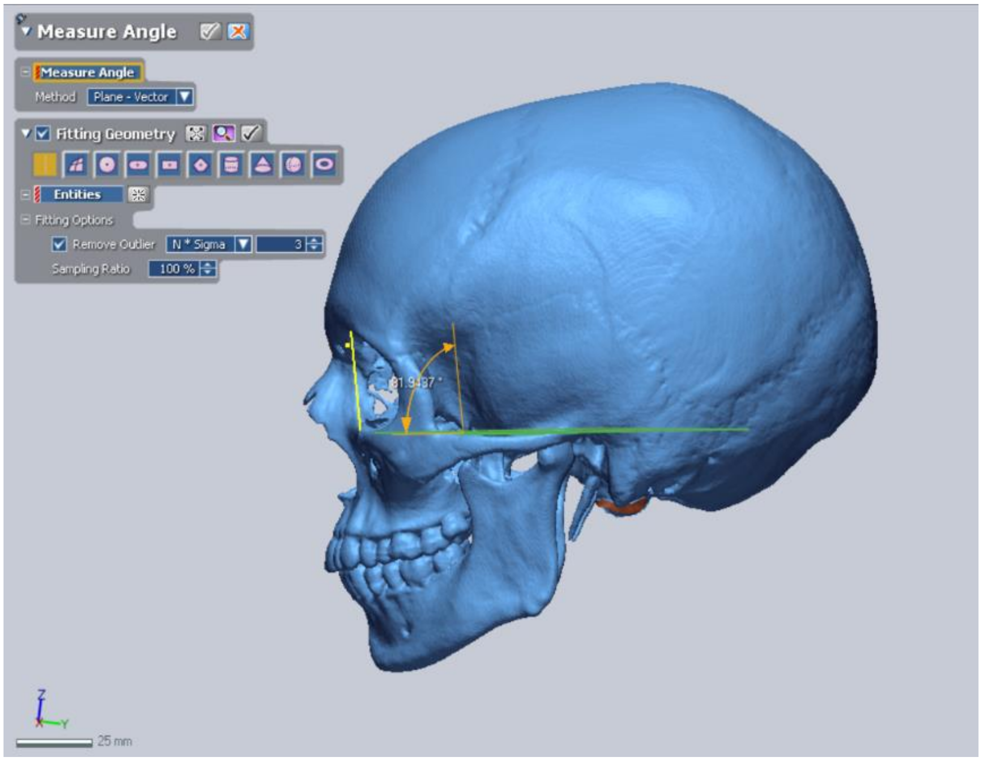

ek-mf-FH

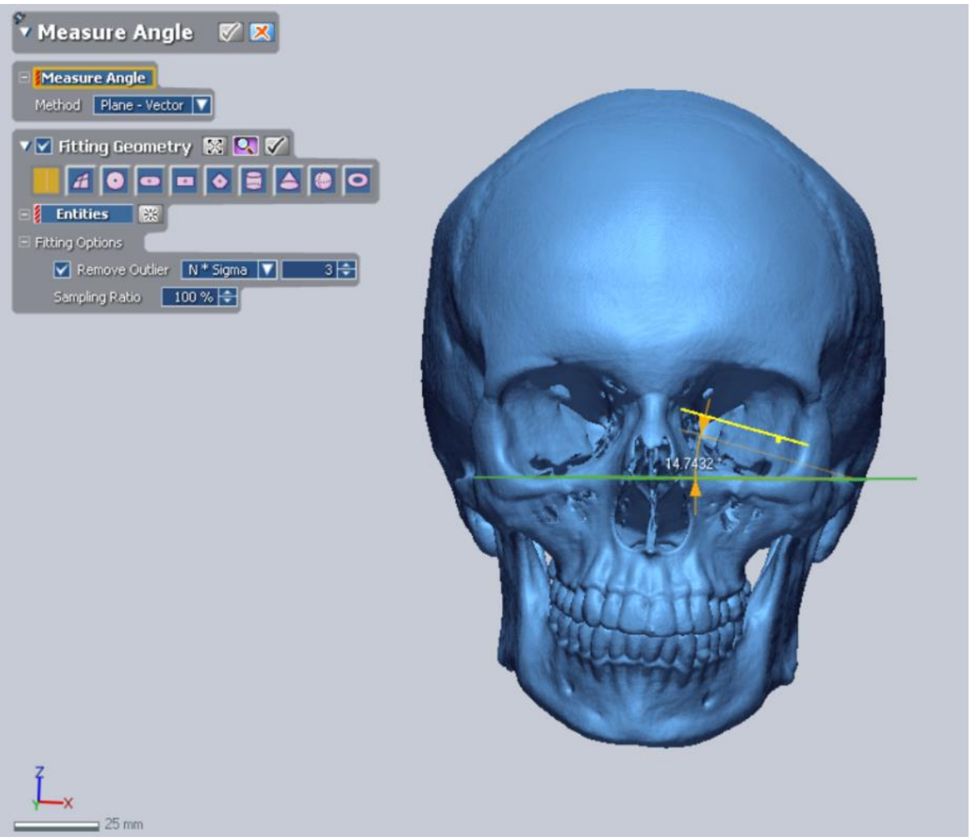

rhi-n-ss

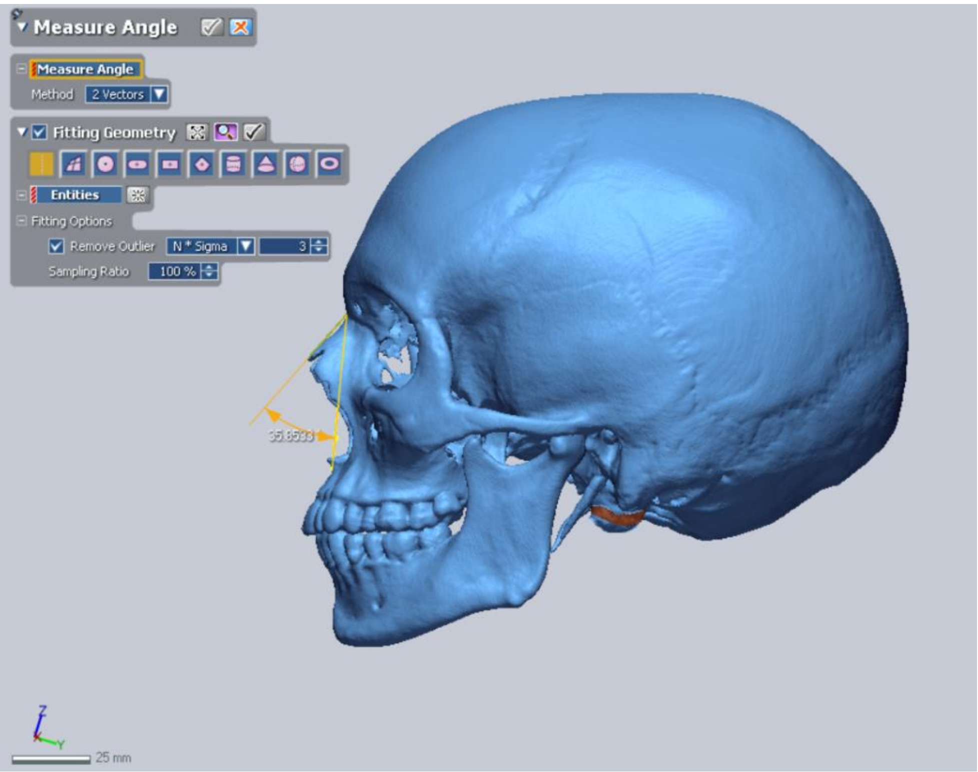

rhi-n-pr

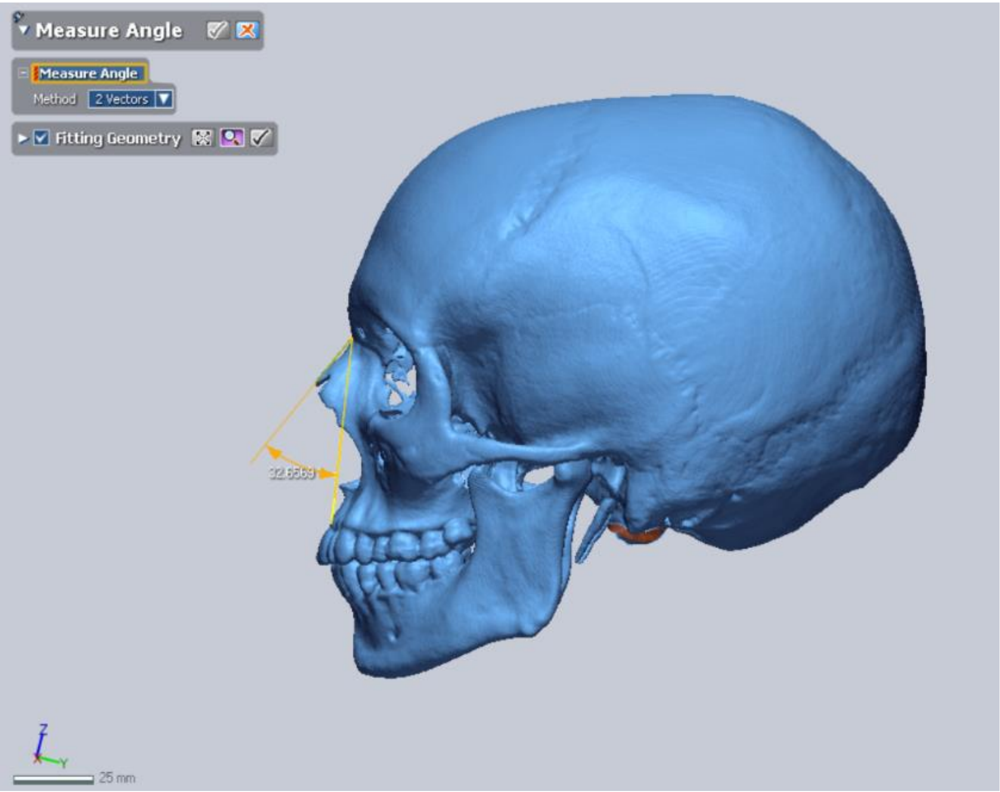

n-mn-rhi

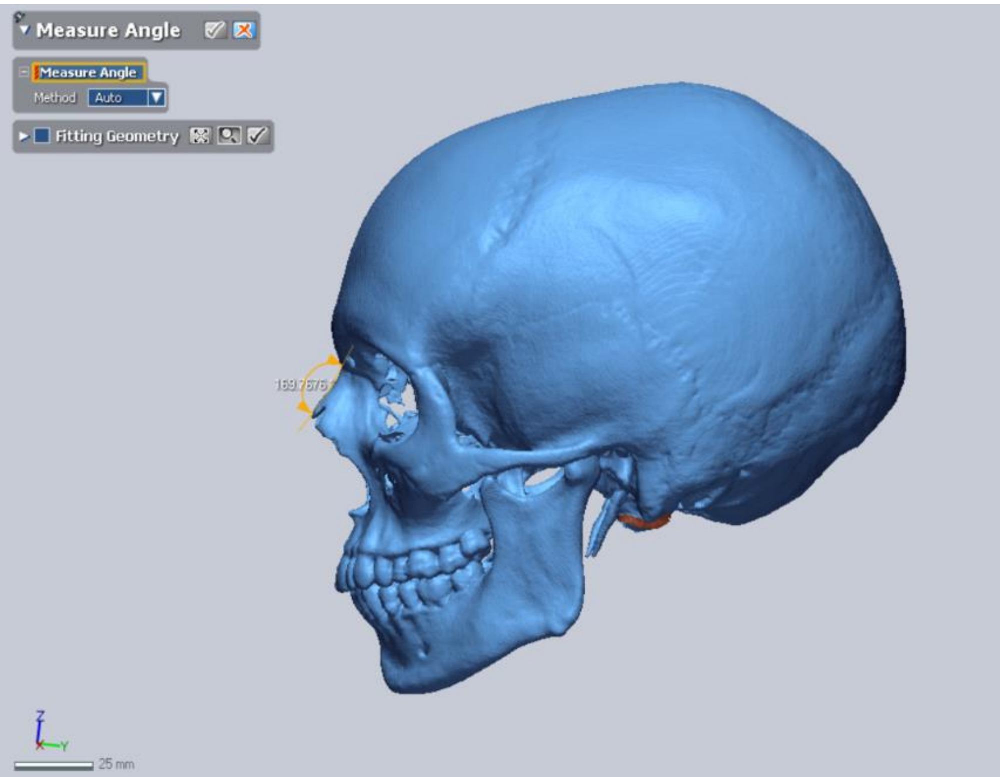

nm-rhi-nm

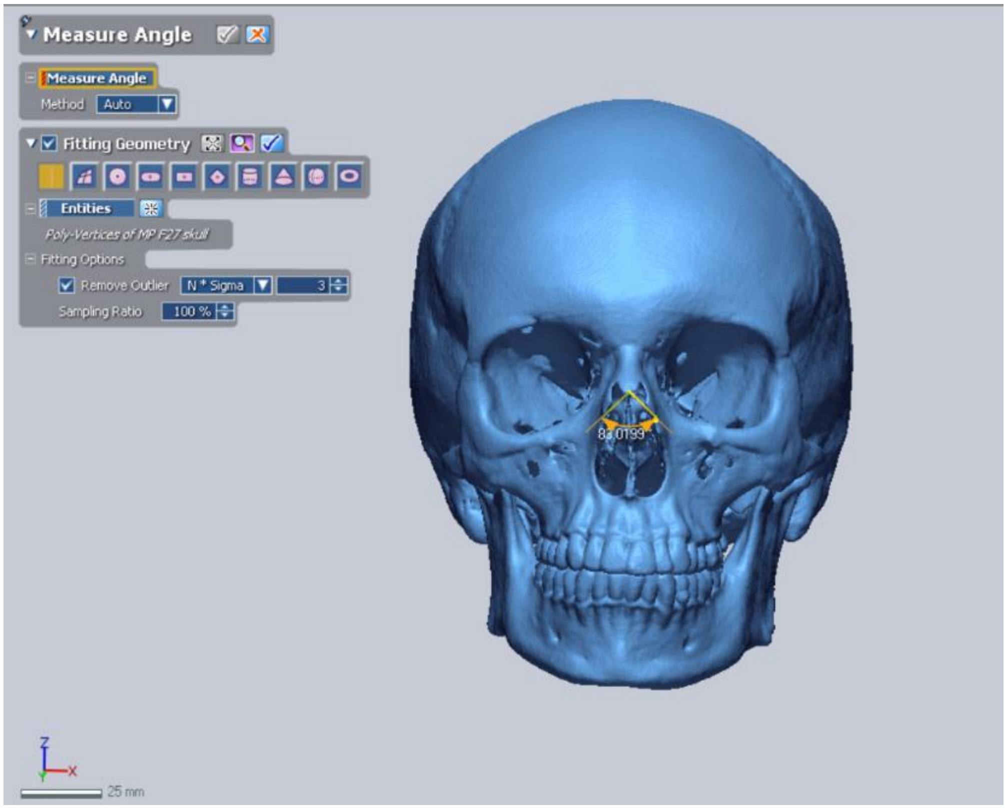

mf-n-mf

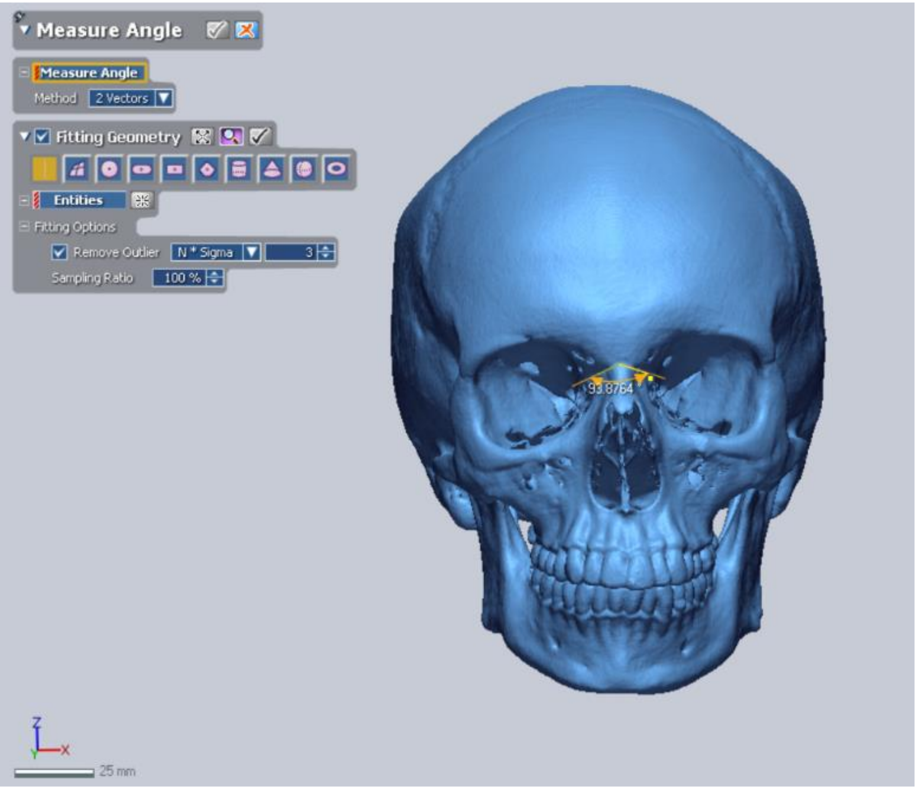

nl-ss-nl

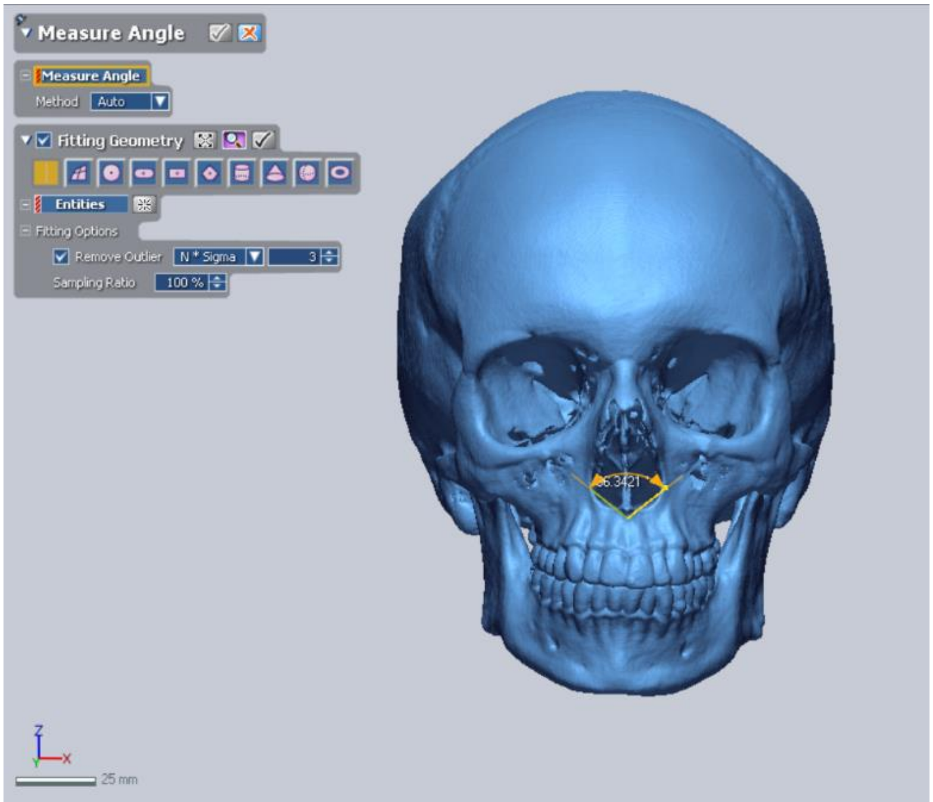

n-rhi-FH

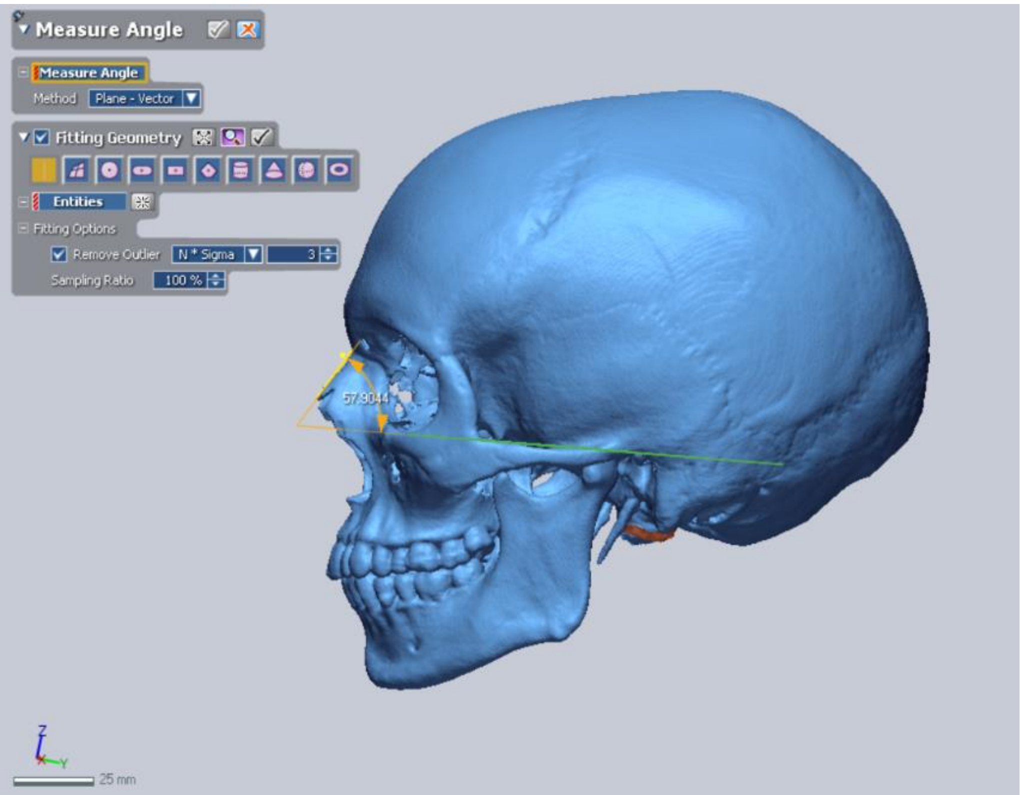

po-ms-ast

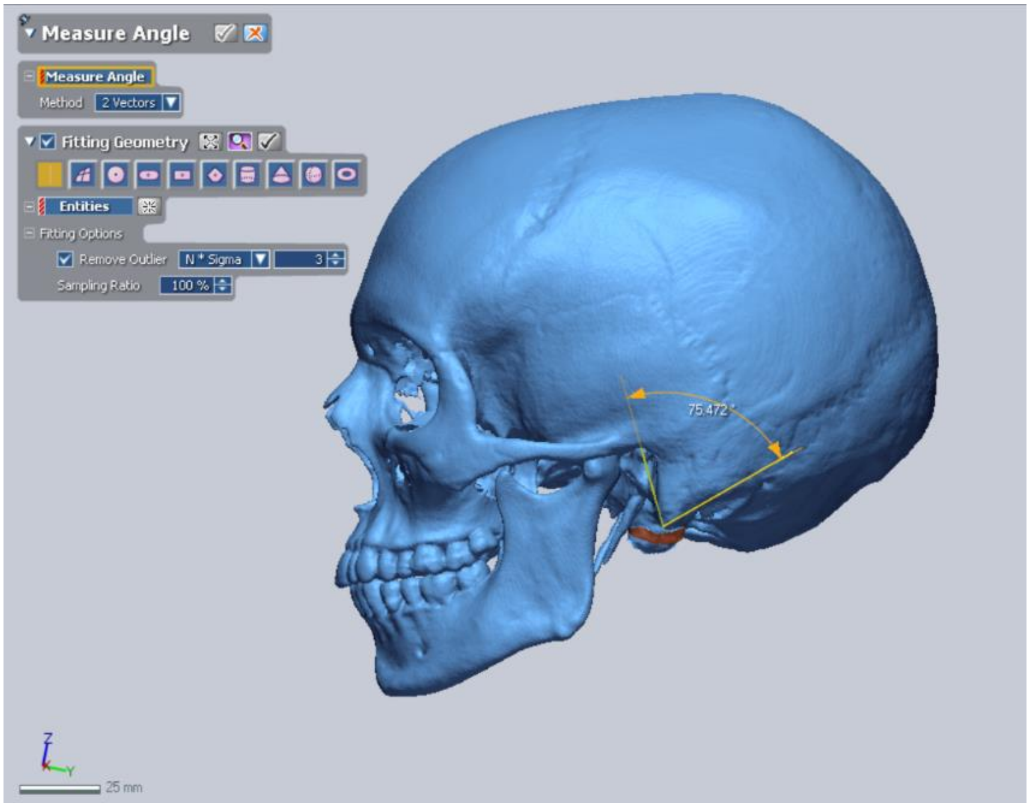

Supplement: Supplementary file 1 [file biology-13-00780-s001.zip › Supplementary File S1.pdf]
