# Supplementary material for: Enhancing Sex Estimation Accuracy with Cranial Angle Measurements and Machine Learning
Source: Biology (Basel). 2024 Sep 29;13(10):780. doi: 10.3390/biology13100780 (PMC11504716; doi:10.3390/biology13100780)
Supplement: Supplementary file 1 [file biology-13-00780-s001.zip › Supplementary File S2.pdf]

Supplementary File S2. Accuracy of the ML models on the separate attribute datasets.

Naïve Bayes

Dataset	Total	Males	Females
27 attr (AI≥0)	87.9 ± 0.5	84.3 ± 0.5	91.0 ± 0.9
19 attr (AI>0)	89.0 ± 0.5	86.7 ± 1.7	90.8 ± 0.3
14 attr (AI≥0.1)	87.2 ± 0.5	82.5 ± 0.9	90.4 ± 0.7
12 attr (AI≥0.3)	86.6 ± 0.9	86.4 ± 2.5	86.8 ± 0.7
9 attr (AI≥0.4)	87.5 ± 0.5	86.5 ± 1.1	88.3 ± 1.1
7 attr (AI≥0.6)	87.3 ± 0.6	85.3 ± 4.1	90.8 ± 0.7
6 attr (AI≥0.9)	87.7 ± 0.5	81.8 ± 0.9	92.6 ± 0.6
4 attr (AI=1)	86.0 ± 0.3	78.1 ± 0.6	92.9 ± 0.5

Logistic regression

Dataset	Total	Males	Females
27 attr (AI≥0)	91.3 ± 0.6	88.5 ± 1.3	93.8 ± 0.7
19 attr (AI>0)	91.8 ± 0.4	89.1 ± 0.8	94.2 ± 0.3
14 attr (AI≥0.1)	88.9 ± 0.6	86.9 ± 1.0	90.7 ± 0.6
12 attr (AI≥0.3)	88.1 ± 0.4	85.9 ± 0.7	90.1 ± 0.3
9 attr (AI≥0.4)	89.4 ± 0.6	85.8 ± 1.2	92.5 ± 0.5
7 attr (AI≥0.6)	87.1 ± 0.9	84.6 ± 4.5	91.0 ± 1.2
6 attr (AI≥0.9)	86.9 ± 0.6	85.0 ± 4.2	90.4 ± 0.8
4 attr (AI=1)	85.8 ± 0.7	83.5 ± 0.7	87.8 ± 0.8

SVM

Dataset	Total	Males	Females
27 attr (AI≥0)	91.9 ± 0.8	89.1 ± 1.4	94.3 ± 0.6
19 attr (AI>0)	92.1 ± 0.4	90.3 ± 1.0	93.7 ± 0.3
14 attr (AI≥0.1)	90.1 ± 0.9	87.3 ± 1.3	92.5 ± 0.5
12 attr (AI≥0.3)	89.6 ± 0.6	86.8 ± 1.0	92.0 ± 0.5
9 attr (AI≥0.4)	89.9 ± 0.6	86.9 ± 1.6	92.6 ± 1.1
7 attr (AI≥0.6)	88.3 ± 0.5	84.7 ± 4.5	91.4 ± 0.5
6 attr (AI≥0.9)	90.0 ± 0.4	87.3 ± 0.4	92.4 ± 0.8
4 attr (AI=1)	86.9 ± 0.7	83.0 ± 1.1	90.3 ± 0.7

CN2

Dataset	Total	Males	Females
27 attr (AI≥0)	83.6 ± 1.3	74.7 ± 1.3	91.2 ± 1.5
19 attr (AI>0)	83.7 ± 1.0	74.5 ± 2.5	91.6 ± 1.2
14 attr (AI≥0.1)	84.1 ± 1.4	76.7 ± 3.2	90.3 ± 1.8
12 attr (AI≥0.3)	84.5 ± 1.8	76.4 ± 2.3	91.4 ± 2.8
9 attr (AI≥0.4)	84.6 ± 1.0	77.2 ± 1.3	91.0 ± 1.8
7 attr (AI≥0.6)	83.8 ± 2.0	75.2 ± 2.2	91.2 ± 2.0
6 attr (AI≥0.9)	84.8 ± 1.2	77.3 ± 2.1	91.2 ± 0.6
4 attr (AI=1)	83.5 ± 0.1	77.0 ± 1.4	89.1 ± 1.1
