# Supplementary material for: Enhancing Sex Estimation Accuracy with Cranial Angle Measurements and Machine Learning
Source: Biology (Basel). 2024 Sep 29;13(10):780. doi: 10.3390/biology13100780 (PMC11504716; doi:10.3390/biology13100780)
Supplement: Supplementary file 1 [file biology-13-00780-s001.zip › Table S1.pdf]

Table S1. Intraobserver measurement error of the landmarks (in mm).

**Landmarks**	**Intraobserver error**
Nasion	0.25
Glabella	0.37
Metopion	0.74
Bregma	0.29
Obelion	0.45
Lambda	0.42
Inion	0.41
Basion	0.24
Opisthion	0.22
Frontomalare-orbitale (right)	0.18
Frontomalare-orbitale (left)	0.18
Maxillofrontale (right)	0.47
Maxillofrontale (left)	0.44
Asterion (right)	0.40
Asterion (left)	0.39
Mastoidale (right)	0.34
Mastoidale (left)	0.28
Porion (right)	0.34
Porion (left)	0.34
Orbitale (left)	0.57
Rhinion	0.18
Subspinale	0.28
Prosthion	0.22
Zygomaxillare (right)	0.35
Zygomaxillare (left)	0.33
Ektokonchion (right)	0.46
Ektokonchion (left)	0.50
Nasolaterale (right)	0.27
Nasolaterale (left)	0.29
Supraorbitale (right)	0.51
Supraorbitale (left)	0.51
Zygoorbitale (right)	0.39
Zygoorbitale (left)	0.43
Midnasale	0.22
Nasomaxillare (right)	0.19
Nasomaxillare (left)	0.18
